# A Toss without a Coin: Information, Discontinuity, and Mathematics in Quantum Theory

**DOI:** 10.3390/e24040532

**Published:** 2022-04-10

**Authors:** Arkady Plotnitsky

**Affiliations:** Literature, Theory and Cultural Studies Program, Philosophy and Literature Program, Purdue University, West Lafayette, IN 47907, USA; plotnits@purdue.edu

**Keywords:** discontinuity, information, Bohr discontinuity, Heisenberg discontinuity, mathematics

## Abstract

The article argues that—at least in certain interpretations, such as the one assumed in this article under the heading of “reality without realism”—the quantum-theoretical situation appears as follows: While—in terms of probabilistic predictions—connected to and connecting the information obtained in quantum phenomena, the mathematics of quantum theory (QM or QFT), which is continuous, does not represent and is discontinuous with both the emergence of quantum phenomena and the physics of these phenomena, phenomena that are physically discontinuous with each other as well. These phenomena, and thus this information, are described by classical physics. All actually available information (in the mathematical sense of information theory) is classical: it is composed of units, such as bits, that are—or are contained in—entities described by classical physics. On the other hand, classical physics cannot predict this information when it is created, as manifested in measuring instruments, in quantum experiments, while quantum theory can. In this epistemological sense, this information is quantum. The article designates the discontinuity between quantum theory and the emergence of quantum phenomena the “Heisenberg discontinuity”, because it was introduced by W. Heisenberg along with QM, and the discontinuity between QM or QFT and the classical physics of quantum phenomena, the “Bohr discontinuity”, because it was introduced as part of Bohr’s interpretation of quantum phenomena and QM, under the assumption of Heisenberg discontinuity. Combining both discontinuities precludes QM or QFT from being connected to either physical reality, that ultimately responsible for quantum phenomena or that of these phenomena themselves, other than by means of probabilistic predictions concerning the information, classical in character, contained in quantum phenomena. The nature of quantum information is, in this view, defined by this situation. A major implication, discussed in the Conclusion, is the existence and arguably the necessity of two—classical and quantum—or with relativity, three and possibly more essentially different theories in fundamental physics.

## 1. Introduction

To begin with mathematics, this article offers a variation on E. Wigner’s theme of “the unreasonable effectiveness of mathematics in the natural sciences” [1]. The article’s view of this unreasonableness is, however, different from that of Wigner. The essence of this unreasonableness, which applies only to quantum theory, specifically quantum mechanics (QM) or, in high-energy regimes, quantum field theory (QFT) (the only quantum theories to be discussed here), may be captured by putting a hyphen in the “un-reasonable”. The hyphen gives the “un-reasonable” its direct meaning, reflecting the main “discontinuity” at stake in this article and interpretations of QM or QFT considered here under the heading of “reality without realism” (RWR)—that between reason and reality in quantum theory. By virtue of this discontinuity, designated here as the “Heisenberg discontinuity”, how the phenomena and the numerical data registered there come about is placed beyond reason and ultimately beyond thought. These data may also be considered as a form of information, which can be treated mathematically in accordance with classical information theory, a mathematical theory of information processing by means of classical physical systems. (Quantum information theory studies information processing by means of quantum systems.) On the other hand, classical physical theories cannot predict these data or information, while the mathematics of quantum theory can—un-reasonably, if one assumes the Heisenberg discontinuity, which precludes a representation or even conception of how these data come about. These predictions are in general probabilistic, which is, however, in accord with what is observed in quantum experiments, as no other predictions are possible, at least thus far.

The main reason for speaking of reason, rather than thought, is I. Kant’s view of reason [*Vernunft*] in his *Critique of Pure Reason* [2], as the greatest capacity of human thought, vis-à-vis understanding [*Verstand*]. “Understanding” deals with *phenomena*, or appearance to human mind, while reason can, in principle, reach *noumena*, or things-in-themselves, which define the ultimate constitution of reality. If one adopts Kant’s scheme, as some interpretations of QM or QFT do, the ultimate reality responsible for quantum phenomena would be noumenal, and thus still reasonable. By contrast, the un-reasonable in question in this article makes it no longer possible for human thought to do so in dealing with this constitution. (Quantum phenomena will be assumed here to be defined by the fact that, in considering them, the Planck constant, *h*, must be taken into account, while putting aside qualifications of this definition, necessary in general but not germane for the present article (e.g., [3] (pp. 37–38)). This is not possible, at least, in RWR-type interpretations of quantum phenomena and QM, or QFT, such as the one adopted in this article, in the strong form of RWR epistemology. The weaker versions of RWR-type interpretations only place the ultimate nature of reality responsible for quantum phenomena beyond knowledge or representation, rather than beyond conception. (As explained below, even this weaker form of RWR epistemology is more radical than Kant’s epistemology.) Accordingly, the capacity of the mathematics of these theories to predict the outcomes of quantum experiments, becomes *un*-reasonable.

I might add that, as I have argued previously [4], the use of mathematics in classical physics or (with qualifications) relativity is, contrary to Wigner’s contention, not unreasonable, let alone *un*-reasonable. As discussed below, the concepts of classical physics are mathematical refinements of the concepts of our general phenomenal intuition. This fact also makes classical physics an essential part of all physics, including quantum theory. In the latter case, however, classical physics is only applicable in considering quantum phenomena, which are observed in measuring instruments and are described, along with the observable parts of these instruments, by classical physics. By contrast, how quantum phenomena come about cannot, at least in RWR-type interpretations defined by the Heisenberg discontinuity, be represented only by classical physics but also by QM or QFT. They can only be predicted, thus, un-reasonably, by the latter, in general probabilistically. Hence, QM or QFT, has, in these interpretations, no physical connection either to the ultimate nature of reality responsible for quantum phenomena or to these phenomena themselves.

By the same token, quantum theory brought about a new type of relationship between mathematics and nature. All modern physics, as a mathematical-experimental science, from R. Descartes and Galileo on, has been defined but by such relationships. However, prior to quantum theory, these relationships had always been representational. As explained below, relativity already exceeded the capacity of our general phenomenal intuition to capture its physical character. Unlike, however, QM or QFT, in RWR-type interpretations, relativity remained a mathematically representational theory, just as is classical physics—most expressly, classical mechanics or classical electromagnetic theory. (Classical statistical physics and chaos theory require certain qualifications of this claim, which are, however, not fundamental, because the same type of representational relationships still ultimately ground these theories). Both classical mechanics or electromagnetism and relativity provide an idealized mathematical representation, say, in Hamiltonian or (more commonly in general relativity) Lagrangian terms. These representations also enable these theories to make predictions, in these cases, ideally exact or deterministic, concerning the future behavior of the systems considered. All such measurements and predictions, and theories themselves, are idealized in modern physics, including in quantum physics, insofar as they disregard many other aspects of reality responsible for the phenomena considered. In quantum theory, however, predictions—at least in RWR-type interpretations—only concerned observed quantum phenomena, but in contrast to (the idealizations of) classical physics and relativity, not the ultimate nature of the physical reality responsible for them. This reality is beyond representation or even conception.

The mathematics of QM or QFT is *continuous*. While, however—in terms of probabilities—related to and relating the information obtained in quantum phenomena, QM or QFT does not, in RWR-type interpretations, represent, is *discontinuous* with, either the emergence of quantum phenomena and the physics of quantum phenomena themselves. These phenomena, which are physically discontinuous with each other as well, are described by classical physics. Thus, this information is classical: it is composed of units, such as bits, that are—or are contained in—entities described by classical physics. There is no other actually available mathematically representable information. This situation defines the conjunction between information and discontinuity as that between the classical and the quantum: information is classical, discontinuity is quantum. On the other hand, classical physics cannot predict this information, while quantum theory can, which in fact grounds quantum information theory as a mathematical theory of information processing by means of quantum systems. Accordingly, at least in RWR-type interpretations, one cannot rigorously speak, as is not uncommon, of a quantum system itself as an information carrier. A quantum system only enables the creation of actual, physically classical information in quantum experiments or their equivalents in nature. In view of the failure of classical physics in predicting this information (described classically but obtained in quantum experiments), this information requires the corresponding mathematical treatment, grounded in the formalism of quantum theory. Quantum information theory provides such a treatment by adopting some concepts of classical information theory and suitably transforming others, for example, by replacing Shannon entropy, a concept defining classical information theory, with von Neumann entropy. (The concept was introduced by von Neumann decades before Shannon in a related but technically different context, as a quantum analogue of Gibbs entropy). It is of course possible that this information or these quantum phenomena could be accounted for by an alternative, conceivably realist and at-bottom deterministic (rather than probabilistic), theory, thus fulfilling the hope of Albert Einstein and many others, often inspired by him. However, it is also possible that no such alternative will be found, especially as concerns the fundamentally probabilistic nature of quantum predictions.

I call the discontinuity between QM and the emergence of quantum phenomena “Heisenberg discontinuity”, because it was introduced by W. Heisenberg along with QM itself, and the discontinuity between QM and the physics of quantum phenomena, “Bohr discontinuity”, because it was introduced as part of N. Bohr’s interpretation of quantum phenomena and QM. The Bohr discontinuity was introduced by Bohr under the assumption of, and as supporting, Heisenberg discontinuity. Combining—as in RWR-type interpretations—both discontinuities precludes QM or QFT from being connected to any physical reality other than by means of probabilistic predictions concerning the information, classical in character, observed in measuring instruments. A major implication, discussed in the Conclusion, is the existence and arguably the necessity of two and possibly more essentially different theories in fundamental physics, the physics that deals with the ultimate constitution of nature. Indeed, with relativity, there are three such theories, or (as each contains several theories) three classes of theories, although as a realist theory, relativity is epistemologically, but not physically or mathematically, akin to classical physics. I shall discuss other quantum discontinuities, including discreteness (a form of discontinuity), beginning with the Planck discreteness, which ushered in quantum theory. Most of them, however, may be seen as grounded in the two forms of discontinuity here defined. Thus, Planck discreteness was so rethought by Bohr. This rethinking replaced the Democritean, “atomic”, discreteness of elemental quantum objects with the discreteness of quantum phenomena, which are physically complex entities (comprised of millions of atoms) observed in measuring instruments.

My argument goes beyond merely rejecting the principle famously expressed as natura non facit saltus, “nature does not make a jump [leap]”, that governed physics and philosophy from Aristotle to Einstein and beyond and that was challenged by quantum theory. Heisenberg discontinuity implies that one can no more claim any form of discontinuity or discreteness, “making jumps”, than continuity, “not making jumps”, or anything else to the ultimate constitution of nature. The reality of this constitution is a reality without realism of any kind, continuous or discontinuous, or a combining of both.

I close this introduction by commenting on my title “a toss without a coin”, a playful expression of the epistemological essence of my argument. Let us consider first a deterministic physical situation in the sense that all predictions considered have either a probability of one or zero. It could be represented by a small ball rolling on a smooth surface. One can observe the ball at any point and (suitably idealizing it as a physical object) represent its motion by the equations of classical mechanics. If one makes, as is always possible, ideally exact measurements of both its position and momentum at the starting point, one can make ideally deterministic predictions concerning it at any point thereafter.

Let us consider next a classical probabilistic situation, a coin toss. The outcome can, in practice, only be predicted probabilistically due to the mechanical complexity of the process, beginning with the motion of the hand tossing it. However, this is still a classically causal process, the outcome of which is determined and could, in principle, be predicted ideally exactly with sufficient technical and computational capacities, which is, as explained later, the meaning of “classically causal”. While self-evident, it is also important that, just as there is always the rolling ball in the first case, there is always the coin as a classical physical object that we observe throughout its trajectory with the head or tail outcome in each case.

Finally, let us consider a quantum experiment, in RWR-type interpretations defined by the combination of the Heisenberg and Bohr discontinuities; say, an emission of an electron from some source resulting in a trace on a silver-bromide screen. The position of the trace can be probabilistically or (by repeating the experiment with the same preparation) statistically predicted by means of QM. However, there is, as things stand now, no way to make ideally exact predictions of the type possible in classical mechanics, regardless of our measuring technology or computational power, because the uncertainty relations would apply even if we had ideal instruments. (In RWR-type interpretations, there is, by Heisenberg discontinuity, no assumption of a classical-like trajectory found, for example, in Bohmian mechanics, although the uncertainty relations are still valid there). No less important is the fact that there is no coin, no analog of a coin, as in principle an observable object: What we observe or predict is only a physically classical trace of the (still presumed) interaction between the electron and the screen, a phenomenon in Bohr’s sense, as a spot produced by a process involving millions of atoms. Thus, there is, metaphorically, a toss, but there is no coin—metaphorically, because it is difficult to think about a toss without an object tossed. An electron is a quantum object, but it cannot be physically observed as such by itself or defined as something that can be tossed to fall in a motion, as the later concept could in principle be conceived by us. This statement still allows one to assume that one can speak of an electron as such—as, while beyond representation or conception, existing by itself, apart from measurement—even in an RWR-type interpretations. This assumption is abandoned in the interpretation adopted here, in which an electron, or any quantum object, is defined as an idealization applicable only at the time of measurement, as considered in detail in [3] (e.g., pp. 71–73). 

The mathematics of QM, enabling this prediction—a mathematics that has no physical connection to the electron by the Heisenberg discontinuity—also has, by the Bohr discontinuity, no physical connection to the observed classical physical object, a spot on the screen. This is because the position of this spot is represented classically within a coordinate system of the instrument. This representation defines the information thus obtained as the only actually possible information obtainable in quantum physics. This information is classical, composed of units such as bits, that are—or are contained in—entities described by classical physics, even though classical physics cannot predict it. The Heisenberg and Bohr discontinuities are strictly quantum. While, however, ultimately defined by the materiality of nature and our technology, beginning with that of our bodies, they are *not physical*. Neither is a discontinuity between two physical entities; the discontinuity between quantum phenomena, defining their discrete nature, is physical, without, in RWR-type interpretations, an assumption of a continuous physical process connecting them. The Bohr and Heisenberg discontinuities are *epistemological*: the first is a discontinuity between the mathematics of quantum theory and the classical data observed, and the second is between this mathematics and how these data come about. In RWR-type interpretations, Heisenberg discontinuity becomes a discontinuity between the corresponding physical reality and our thought itself. The mathematics of classical physics or relativity connects to—and in this sense has continuity with, correlatively—both the observed phenomena (which can, for all practical purposes, be identified with the objects considered) and with how these phenomena come about. Both forms of continuity are abandoned in RWR-type interpretations of quantum phenomena and quantum theory.

The remainder of this article proceeds as follows. The next two sections consider Heisenberg and Bohr discontinuity, respectively. Section 4 offers a commentary on the nature and multiplicity of fundamental theories in physics, and the possibility—or impossibility—of their unification, in view of this article’s argument.

## 2. Heisenberg Discontinuity: From Planck to Heisenberg, with Abstract Mathematics

The denomination “modern” has been commonly used to refer to different historical periods in mathematics and physics. In mathematics, “modern” tends to refer to the mathematics that had emerged in the nineteenth century, with such figures as K. F. Gauss, H. Abel, and É. Galois. In physics, on the other hand, it refers to all mathematical-experimental physics, beginning with that of Galileo and R. Descartes, which used the mathematics (algebra, analytic geometry, and calculus) developed around their time. This is fitting because this physics emerged along with and shaped the rise of modernity, making it scientific. Modernity is defined by several interrelated transformations, sometimes known as revolutions, although each took a while. Among them are scientific (defined by the new cosmological thinking, beginning with the Copernican heliocentric view of the Solar System, and the introduction of physics as the mathematical-experimental science); industrial, or more broadly, technological (defined by the primary role of machines in industrial production and beyond); philosophical-psychological (defined by the rise of the concept of the individual human self, beginning with Descartes’s concept of the Cogito); economic (defined by the rise of capitalism); and political (defined by the rise of Western democracies). One might add to this list the mathematical revolution, rarely discussed as such, although considered as part of the scientific revolution. If, however, modernity is scientific, as it is assumed to be, it is also because it is mathematical. As M. Heidegger argued in commenting on Galileo and Descartes, “modern science is experimental because of its mathematical project” [5] (p. 93). The term “classical physics” was introduced (in the 1920s), following the discovery of relativity and quantum physics, still considered modern by virtue of their mathematical-experimental character.

On the other hand, one of the defining aspect of modern mathematics was the aim of making mathematics independent from its relations to nature and from physics, as well as from our daily phenomenal intuition and concepts. It is this double independence that gives to modern mathematics its fundamentally abstract character. It is significant, including in the context of this article, that this drive for independence became eventually connected to “the crisis of continuity”. According to J. Gray, in speaking of what he calls mathematical “modernism” (the twentieth-century culmination of modern mathematics): “This is the widespread feeling among mathematicians around 1900, documented in many sources, that the basic topic of analysis, continuity, was profoundly counterintuitive. This realization marks a break with all philosophy of mathematics that present mathematical objects as idealizations from natural ones” [6] (p. 20). Gray’s claim requires qualifications. First, mathematics separated—abstracted—itself from representing natural objects and physics, as well as from our daily phenomenal world much earlier. In some respects, it has done so even in ancient Greek mathematics. This separation became pronounced with the rise of algebra, especially the study of algebraic equations, at the outset of modernity. It is true, however, that both geometry and analysis had kept closer relationships with representing nature in physics, and thus with our daily thinking and its representation of nature. This connection is defined by the fact that classical physics mathematically idealizes this daily-life representation of natural objects and motions. In any event, by 1900 mathematics had reached the stage of breaking with the connections to natural objects and physics in most areas of mathematics, including geometry and analysis. G. Cantor’s introduction of set theory and his investigations of the nature of mathematical infinity, culminating in the continuum hypothesis, was arguably the most significant development leading to “the crisis of continuity” invoked by Gray. Cantor’s rethinking of the nature of mathematical infinity radically and irrevocably separated the concept of continuity in mathematics from our phenomenal intuition of continuity. Quantum theory, beginning with M. Planck’s discovery of it in 1900, was about to contribute to this crisis. QM, especially in RWR-type interpretations, established a new type of relationship between mathematical continuity of the theory and physical discontinuity, by combining the Heisenberg and Bohr discontinuity. In these interpretations, QM limited the relationships between its mathematics and quantum phenomena to probabilistic predictions of the information observed in measurements and described classically.

This divorce of modern mathematics—including that of continuity—from both the representation of natural objects and our general phenomenal intuition was stressed by H. Weyl. Weyl made this point in his 1918 *The Continuum* [7], followed closely by his 1918 classic *Space Time Matter* [8], by which point quantum theory (although not yet QM) entered his thinking [8] (p. 23). According to Weyl (referring to Cantor’s investigation of the concept of the continuum that led Cantor to his continuum hypothesis): “the conceptual world of mathematics is so foreign to what the intuitive continuum presents to us that the demand for coincidence between the two must be dismissed as absurd” [7] (p. 108). “Coincidence” is, however, not the same as “relations”, which are unavoidable, at least insofar as it is difficult to think of continuity spatially apart from our phenomenal intuition. On the other hand, it was possible to define the continuum rigorously algebraically, or so it appeared. The situation was changed again, even more dramatically, by Gödel’s incompleteness theorems of 1931 and eventually P. Cohen’s proof of the undecidability of the continuum hypothesis in the 1960s. These findings made us realize not only that we do not but also that we cannot know how a continuous line, straight or curved (which does not matter topologically), is constituted by its points. We might have a phenomenal intuition of continuity, but we cannot rigorously think of it mathematically as comprised of points. Quantum theory eventually suggested that continuity, of any kind, may not be found in nature at the ultimate level of its constitution either; but then, neither may be discontinuity of any kind.

Ironically, however, with relativity and quantum theory, physics was able to take advantage of this divorce between mathematics and our general phenomenal intuition, and with QM between mathematics and nature. Weyl added to his statement cited above: “Nevertheless, those abstract schemata supplied us by mathematics must underlie the exact sciences of domains of objects in which continua play a role” [7] (p. 108). This comment was made primarily with Einstein’s relativity in mind, as Weyl’s next book, *Space Time Matter* [8], devoted to relativity, was in the works. The abstract of schemata of modern mathematics used in relativity and then QM, that of Riemannian geometry and that of Hilbert spaces (of finite and infinite dimensions) over ℂ, are divorced from our phenomenal intuition. There was, however, a crucial difference, due to the fundamental role of discontinuity in QM, even though both theories use continuous mathematics.

In relativity this (idealized) representation of the workings of nature was possible, because the break from our phenomenal intuition need not entail a divorce from a representation of nature, even physical, let alone mathematical. The latter is, in principle, possible in the absence of a physical ontology, even in QM in certain interpretations, although not the one adopted in this article. Relativity was, however, a radical departure from classical physics in this regard, because the relativistic law of addition of velocities (defined by the Lorentz transformation) in special relativity,$\;s=\frac{v+u}{1+{\left(vu/c\right)}^{2}}$, for collinear motion (*c* is the speed of light in a vacuum), is beyond our general phenomenal intuition of motion. We cannot conceive of this type of motion, which is thus no longer a mathematical refinement of a daily concept of motion as the classical concept of motion is. Relativity was the first physical theory that defeated our ability to form a phenomenal conception of an elementary individual physical process, although the concept of electromagnetic field (which also essential in special relativity) posed difficulties in this regard as well. Relativity theory was a form of discontinuity between nature and our phenomenal intuition, rather than nature and the mathematics of relativity; the theory allowed for a mathematized representation of the physical reality considered.

In general relativity, this representation is defined by the Riemannian geometry of space, which requires tensor calculus for establishing the field equations of the theory, such as Einstein’s field equations, generally in the form:

$$G_{\mu \nu }+\Lambda g_{\mu \nu }=\kappa T_{\mu \nu }$$

$(G_{\mu \nu }$ is the Einstein tensor; $G_{\mu \nu }=R_{\mu \nu }-\frac{1}{2}\;Rg_{\mu \nu },\;R_{\mu \nu }$ is the Ricci curvature tensor, a symmetric second-degree tensor that depends only on the metric tensor and its first and second derivatives; R is the scalar curvature; $T_{\mu \nu }$ is the stress energy tensor; Λ is the cosmological constant, and $\kappa =\frac{8\pi G}{c^{4}}\;\approx 2.077\times 10^{-43}N^{-1}$ is the Einstein gravitation constant, with *G* in the Newtonian gravitational constant). Deceptively elegant in appearance, this equation unfolds into a system of ten nonlinear, hyperbolic-elliptic partial differential equations. This is, mathematically, a far cry from anything based in our general phenomenal intuition, which also cannot imagine such geometrical objects as three-dimensional surfaces, say, a three-diemansional sphere, which are considered in the general-relativistic cosmology. This mathematics is a product of the divorce, defining modern mathematics, between mathematics and this intuition, which are more closely connected in classical physics. Infinitesimally, the spaces considered are geometrically Euclidean, in accordance with Riemann’s concept of manifold, mathematically defining a Riemannian physical space. Accordingly, physically, special relativity applies in a local or infinitesimal neighborhood of each point, which gives the corresponding spacetime the Minkowskian metric. Physically, this metric corresponds to the fact that all reference frames that are in free fall are equivalent. The concept of spacetime was introduced by H. Minkowski in 1908 and was not initially used by Einstein, who was indeed ambivalent toward it at first, but eventually embraced it. While uncontroversial as a mathematical tool, as a physical concept, the concept of spacetime involves complexities and its status as a physical concept has been debated. In any event, unlike a Riemannian geometry of space, it is not germane in the present context, and need not be considered here. My point is that while using mathematical and physical concepts that defeat our general phenomenal intuition, relativity—special and general—remains a realist and classically causal, in fact deterministic, theory. It does not contain anything like either Heisenberg or Bohr discontinuity.

The theory, beginning with special relativity, did introduce new features of measurement and changed the role of measuring instruments (rods and clocks), making space and time defined by them, rather than something assumed to be pre-existing and then measured by rods and clocks, as were Newtonian absolute space and time. Nevertheless, just as classical mechanics, special relativity predicted the outcomes of measurements on the basis of its mathematical formalism, which in the first place represented how these outcomes come about. Most essentially, this was possible because as in classical mechanics, one could for all practical purposes assume that, in Bohr’s words, “the phenomena concerned may be observed without disturbing them appreciably”, as against quantum phenomena [9] (v. 1, p. 53).

By contrast, QM—at least in RWR-type interpretations—is disconnected from both the physical emergence of quantum phenomena, no longer represented by QM, defined here as Heisenberg discontinuity, and the observed quantum phenomena, represented by classical physics and not QM, defined here as Bohr discontinuity. QM, however, related quantum phenomena probabilistically, which classical physics could not do. This incapacity led to the rise of quantum theory and eventually to the development of QM. These probabilistic predictions were, moreover, only possible by using rules added to the formalism rather than being part of it, such as Born’s rule. Born’s rule relates, essentially, to using complex conjugation, complex quantities of the QM formalism to real numbers corresponding to the probabilities of quantum events (technically, to probability densities). Representations in terms of (mathematized) physical concepts and the corresponding physical ontologies are, thus, still necessary in quantum physics, in RWR-type interpretations: they are necessary in dealing with the reality observed in measuring instruments, defining quantum phenomena. On the other hand, a knowledge or even conception of the ultimate nature of reality responsible for quantum phenomena is no longer offered, or assumed to be possible to offer, in these interpretations.

This concept of reality and the corresponding interpretations of quantum phenomena and quantum theory, both linked to the Heisenberg and Bohr discontinuities, are designated here as “reality-without-realism” (RWR) and RWR-type interpretations, under the overall heading of the RWR view. The concept of RWR and the RWR view was discussed by this author previously, most extensively in [3] (which contains further references), and it will only be briefly sketched here. This concept presupposes general concepts of reality and existence, assumed to be primitive concepts and not given analytical definition. By reality I understand that which is assumed to exist without making any claims concerning the character of this existence. Such claims define realism, which, in most understandings of the term, assumes the possibility of forming an (idealized) representation or at least a conception of the reality responsible for the phenomena considered. The absence of such a claim allows one to adopt the RWR view by placing a given reality or part of it beyond representation or knowledge, designated here as “the weak RWR view”, or conception, designated as “the strong RWR view”, which I adopt here, as did Bohr in his ultimate interpretation, developed around 1937. By contrast, Heisenberg, who appears to have held the weak RWR view at the time of his discovery of QM, eventually shifted to a more realist view. In the form of realism he adopted, however, giving it a Platonist bent, the ultimate nature of the reality responsible for quantum phenomena could only be represented mathematically in the absence of physical concepts, at least as they are ordinarily understood, say, in classical physics or relativity. Bohr discontinuity could still be retained, and it was by Heisenberg. Heisenberg discontinuity implies Bohr discontinuity, but the latter is possible without Heisenberg discontinuity.

There is still the question of whether the RWR view, weak or strong, only:

(A) characterizes the situation as things stand now, while allowing that quantum phenomena or whatever may replace them will no longer make this assumption and thus RWR-type interpretations viable, thus reverting to a realist view; or
(B) reflects the possibility that this reality will never become available to thought.

Logically, once (A) is the case, then (B) is possible, but is not certain. There do not appear to be any experimental data compelling one to prefer either. (A) and (B) are, however, different in defining how far our mind can, in principle, reach in understanding nature. This is the main reason to distinguish them, although most of my argument in this article applies to both. As discussed in the next section, Bohr at least assumed (A), and some of his statements suggest that he might have entertained (B). The qualification “as things stand now” applies, however, to (B) as well, even though it might appear otherwise given that this view precludes any conception of the ultimate constitution of the reality responsible for quantum phenomena not only now but also ever. It applies because a return to realism in quantum theory is possible, if quantum theory, as currently constituted, is replaced by an alternative theory that requires a realist interpretation. This might make the strong (or weak) RWR view obsolete even for those who hold it, with quantum theory in place in its present form. I shall make an additional assumption, not found in Bohr, that the concept of quantum object, beginning with elementary particles, such as electrons or photons, is an idealization only applicable at the time of observation, as considered in detail in [3]. Nature has no quantum objects; they are idealizations created by us in our interaction with nature, in the present view, again, as idealizations applicable only at the time of observation. This view implies that it is only possible to speak of the same quantum object, such as an electron or photon, in two successive observations as a provisional idealization, an indealization, moreover, only permissible in low-energy (QM) regimes but not in high-energy (QFT) regimes, as explained in [3] (pp. 71–73, 283–285). 

Beginning with its discovery, at the intersection of electromagnetism, thermodynamics, and kinetic atomic theory, by Planck, in his black body radiation law, in 1900, and throughout “the old quantum theory” (preceding QM), quantum theory has always been a probabilistic or statistical theory. It has also always been a theory entailing defining forms of discontinuity and discreteness, such as Planck’s discontinuous quanta of radiation, Einstein’s concept of a photon, Bohr’s quantum jumps, and ultimately Bohr’s concept of (quantum) phenomena as irreducibly discrete relative to each other. On the other hand, its character as a form of information theory only emerged with QM, although it took over half a century to realize and express this character in quantum information theory. At the same time, quantum discontinuity acquired new and more radical forms at stake in this article, the Heisenberg and Bohr discontinuities, combined in RWR-type interpretations. Nature has no information either, only we do. By Bohr discontinuity, however, QM is physically disconnected from this information, represented classically (in terms of classical bits) as contained in measuring instruments, as well: QM is only linked to this information mathematically by probabilistically predicting it.

In this view, a pure qubit state (a state vector in the Hilbert-space formalism) |ψ⟩=α |0⟩+β|1⟩ is not a unit of information, any actual physical information, obtained from any physical state of the quantum system considered independently. As a physical system, any quantum system is beyond knowledge or even conception in RWR-type interpretations. A qubit is an element of the formalism that allows us to predict—with the help of Born’s rule (α and β are probability amplitudes)—the probabilities of obtaining new information, with value 0 or 1, as an outcome of a future measurement on the basis of the information obtained in a previous measurement.

The same situation is obtained for any quantum system and variables (discrete or continuous), say, as treated, in nonrelativistic quantum regimes, by Schrödinger’s (time dependent) equation:

$$i\hbar \frac{d}{dt}\;\left|\Psi \left(t\right)\rangle =H\right|\Psi \left(t)\right\rangle .$$

(Ψ is the wave function and *H* the Hamiltonian). The equation may look like an equation of a continuous (wave) motion and was initially assumed by Schrödinger to be. However, it is not, even in most realist interpretations. In RWR-type interpretations, it only enables one to predict (with the help of Born’s rule) the probabilities of possible future measurements, on the basis of the initial measurement or preparation, without offering and even precluding a representation of the independent behavior of the system in space and time. The formalism, beginning with qubits, also “encodes”, which is again to say, allows one to predict, such quantum effects as interference, observed in the double-slit experiment, or quantum correlations, observed in the Einstein–Podolsky–Rosen (EPR)-type experiments. This situation is, in RWR-type interpretations, no different in high-energy, relativistic quantum regimes and the formalism of QFT, beginning with Dirac’s equation. If anything, these regimes introduce still more radical epistemological features [3] (pp. 273–306).

In view of these considerations, the ground for the use of probability change vis-à-vis classical physics (when the latter uses probability). In Bohr’s words:

It is most important to realize that the recourse to probability laws under such circumstances is essentially different in aim from the familiar application of statistical considerations as practical means of accounting for the properties of mechanical systems of great structural complexity [in classical physics]. In fact, in quantum physics we are presented not with intricacies of this kind, but with the inability of the classical frame of concepts to comprise the peculiar feature of indivisibility, or “individuality”, characterizing the elementary processes.

[9] (v. 2, p. 34)

This “feature” reflects the essential discontinuity of quantum phenomena and QM, as defined the Heisenberg and Bohr discontinuities, which also makes any two quantum phenomena discrete relative to each other (which is different from the particle-like discreteness of quantum objects). In commenting on the invention of QM, Heisenberg suggested the reasons for Heisenberg discontinuity:

It is not surprising that our language should be incapable of describing the processes occurring within the atoms, for … it was invented to describe the experiences of daily life, and these consist only of processes involving exceedingly large numbers of atoms. Furthermore, it is very difficult to modify our language so that it will be able to describe these atomic processes, for words [when they describe things] can only describe things of which we can form mental pictures, and this ability, too, is a result of daily experience. Fortunately, mathematics is not subject to this limitation, and it has been possible to invent a mathematical scheme—the quantum theory—which seems entirely adequate for the treatment of atomic processes [in terms of predicting the outcomes of quantum experiments]; for visualizations, however, we must content ourselves with … incomplete analogies [such as] the wave picture and the corpuscular picture.

[10] (p. 11)

One might contest the view that “words can only describe things of which we can form mental pictures”, although it may be true that they can only describe things that allow for representations. This qualification would not, however, undermine Heisenberg’s main point, which is as follows: By virtue of our evolutionary biological or neurological constitution, our language or thinking—at least our general phenomenal thinking but possibly any thinking, except perhaps mathematical one—may not be capable of representing, or even conceiving, the ultimate microscopic constitution of nature, because atoms, let alone elementary particles, are extremely small. (Quantum objects—that is, objects exhibiting quantum behavior—may be macroscopic, as are Bose–Einstein condensates, but their quantum behavior is still defined by their ultimate microscopic constitution.) Our mathematics, however, including that which is highly abstract in character (in QM essentially that of Hilbert spaces over ℂ), while still the product of this neurological constitution, is capable of predicting quantum phenomena. Heisenberg’s statement in principle allows for the possibility of a mathematical representation of the ultimate nature of the reality responsible for quantum phenomena in the absence of physical concepts. In contrast to Bohr, Heisenberg, as I said, eventually came to think this to be possible, although, arguably, not at the time of this statement in 1929–1930, when he held a weak RWR view. A few qualifications are in order.

First, at least in RWR-type interpretations, although heuristically useful, all visualizations used in QM or QFT are not only incomplete but are also provisional. In these interpretations, no representation of any kind, visualizable or not, or even conception of quantum objects and processes is possible.

Secondly, that Heisenberg found a mathematical scheme that could predict the data in question was as fortunate as that mathematics is free of this limitation. We have been equally fortunate to invent the mathematics of classical physics and relativity, even though in these cases—especially for classical mechanics—we were helped by our general phenomenal intuition, which classical mechanics mathematically refined. By contrast, the formalism of QM is entirely abstracted from this intuition. As noted, relativity already posed insurmountable limits for our phenomenal intuition. Mathematically, however, it remained a representational, realist theory.

Thirdly, although QM is fully in accord with quantum experiments insofar as no predictions other than probabilistic ones are possible, the “adequacy” of QM, especially in this interpretation, has been questioned. Einstein, who led the way of this questioning, had never accepted that a mathematical scheme only capable of such predictions is “entirely adequate” in fundamental physics. This adequacy remains under debate, notwithstanding enormous successes of QM and QFT as probabilistic theories, probabilistic even in dealing with the most elementary quantum objects, such as elementary particles.

In RWR-type interpretations, no two experimentally observed quantum phenomena are assumed to be connected physically, specifically in terms of the motion of quantum objects, as the concept of motion is understood in classical physics and relativity, or possibly any concept of motion we can form. As Heisenberg said later: “There is no description of what happens to the [quantum] system between the initial observation and the next measurement” [11] (p. 47). Nobody has ever observed a moving quantum object as such; one can only observe traces left by their interaction with measuring instruments, such as spots on photographic plates. The same, it follows, would apply to the word “happen” or “system”, or any word we use, whatever concept it may designate, including reality, although when “reality” refers to that of the RWR-type, it is a word without a concept attached to it. As Heisenberg added: “But the problems of language are really serious. We wish to speak in some way about the structure of the atoms and not only about ‘facts’—the latter being, for instance, the black spots on a photographic plate or the water droplets in a cloud chamber. However, we cannot speak about the atoms in ordinary language” [11] (pp. 178–179). Nor is it possible in terms of ordinary concepts, from which ordinary language is indissociable, or in the RWR view, even in terms of physical concepts, assuming that they can be fully dissociated from ordinary concepts.

As indicated, there remains a question of whether the mathematical scheme of QM or QFT, or any mathematical scheme, can represent what “happens to the [quantum] system between the initial observation and the next measurement”, in the absence of a verbal description or even physical concepts, at least as they are ordinarily understood, say, in classical physics or relativity. This possibility was, as noted, entertained by Heisenberg at the time of the second statement just cited (e.g., [11] (pp. 145, 167–186)), but not—at least not expressly—at the time of his discovery of QM. There appears to be, however, no special reason to assume that human mathematical thinking should, as still human, be able to conceive of the workings of nature on these scales, such as the Planck scale. It may not relate to these workings otherwise, including in terms of probabilistic predictions, still possible in QM or QFT. Our mathematical and (in experimental physics) technological thinking has done reasonably and even surprisingly well and might continue to do well. But how far could our mathematics reach, even if we use the universe itself as the source of experiments? This mathematics is only human, a product of our evolutionary biological and neurological constitution, while the Universe that created evolution and us is not human, even if it is not governed by anything divine either.

On the other hand, information, mathematical or not, *is* human. For reasons that must already be apparent and will be spelled out further in the remainder of this article, I shall propose that Heisenberg’s thinking that led him to his discovery of QM suggested that QM may be understood as, correlatively, a fundamentally probabilistic and fundamentally informational theory, and thus as a form of quantum information theory. One could not, of course, say that Heisenberg’s matrix mechanics was envisioned by him or was at the time a form of quantum information theory in the current meaning of the term. It was quantum-informational in spirit. Conversely, quantum information theory may be seen as Heisenbergian in spirit, if one assumes the RWR view of quantum information theory and, hence, both Heisenberg and Bohr discontinuity there, although Bohr discontinuity was at most only implicit in Heisenberg’s argumentation in his derivation of QM.

Bohr brought Heisenberg discontinuity into the foreground as the defining feature of QM (at least in the corresponding interpretation) in his initial assessment of Heisenberg’s “new quantum mechanics”. More accurately, he made this assessment after the theory was given its full-fledged form as matrix mechanics by M. Born and P. Jordan, but before Schrödinger’s introduction of his wave mechanics in 1926. Schrödinger based his approach on realist principles and the assumption of the continuous (wave-like) nature of the ultimate reality responsible for quantum phenomena, expressly in juxtaposition to matrix mechanics as a discontinuous theory. It might, accordingly, appear, and had initially appeared, remarkable that he arrived at a mathematical scheme that made the same predictions. Indeed, both schemes were quickly shown to be mathematically equivalent. Ultimately, it was not so surprising, because in his derivation of his scheme, specifically his famous equation, Schrödinger made several mathematical moves—guesses—that had no justification in his physical programs [3] (pp. 145–166). Heisenberg, by contrast, consistently followed his physical principles in developing matrix mechanics. Bohr’s assessment was in accord with the same principles as well:

In this theory the attempt is made to transcribe every use of mechanical concepts in a way suited to the nature of the quantum theory, and such that in every stage of the computation only directly observable quantities enter. In contrast to ordinary mechanics, *the new quantum mechanics does not deal with a space–time description of the motion of atomic particles*. It operates with manifolds of quantities which replace the harmonic oscillating components of the motion and symbolize the possibilities of transitions between stationary states in conformity with the correspondence principle. These quantities satisfy certain relations which take the place of the mechanical equations of motion and the quantization rules.

[9] (v. 1, p. 48; emphasis added)

“Directly observable quantities” echoes Heisenberg’s famous statement opening his first paper on QM by announcing that it will deal with the “relationships between quantities which in principle are observable” [12] (p. 263; emphasis added). This statement has often been misunderstood on empiricist lines, in part by disregarding the word “relationships”. Heisenberg’s new mechanics did not deal only with “quantities which in principle are observable”, although it was aimed strictly at predicting such quantities and *only* them, which is very different. The theory was essentially concerned with quantum objects, unobservable in themselves, but having observable effects on measuring instruments. As such, it departed from most forms of empiricism, such as that of E. Mach, who argued that physics should only be concerned with what can be observed. This view was equally rejected by Einstein, who believed that one can only approach physical reality through (mathematized) concepts [13] (p. 47). Heisenberg, in fact by virtue of Heisenberg discontinuity automatically, also rejected Mach’s view as concerns the ultimate nature of the reality responsible for quantum phenomena. Heisenberg’s epistemology, defined by Heisenberg discontinuity, was of course more radical than that of Einstein, who never gave up realism.

Heisenberg followed Bohr’s 1913 theory, the first step in this direction. Bohr’s theory retained the classical (orbital) behavior of electrons in the so-called stationary (constant-energy) state, a behavior already in conflict with classical electrodynamics. However, now in conflict with both classical electrodynamics and classical mechanics, Bohr’s theory postulated the discontinuous transitions (“quantum jumps”) of electrons from one energy level to another, as they absorbed or emitted Planck’s energy quanta. The radical nature of Bohr’s move, which changed the nature of fundamental physics, was defined by the fact that no mechanical description of these transitions was offered or even assumed. Heisenberg then abandoned the idea (which ran into difficulties by the 1920s) of representing electrons as orbiting the atomic nuclei in the stationary state between which quantum jumps occurred. In a way, in Heisenberg’s theory there were only “quantum jumps” between the states of a quantum system, such as an electron. Indeed, the term “jump” is misleading as suggesting some representation of what happens. Electrons do not jump; quantum states (as physical states) discontinuously change, and no representation of how they do so was available. What was responsible for these changes was assumed to be real, but this reality was deemed to be beyond representation, in accord at this stage with the weak RWR view. The strong RWR view was already intimated, however, insofar as no concept of how these transitions appeared to be possible to form either.

“Directly observable quantities” in question were formed or could be represented by classical (bits of) information observed in measuring instruments. The “manifolds of quantities” invoked by Bohr in his statement above were elements of Heisenberg’s matrices, new variables of his mechanics. These matrices contained both quantities: those taken from previous observations, which were real quantities (actual numbers) and purely mathematical elements, which were complex mathematical entities.

The “relations which take the place of the mechanical equations of motion and the quantization rules” of the old quantum theory are formally classical, such as Hamiltonian, equations of motion. In the old quantum theory, such as Bohr’s 1913 atomic theory, these equations, while still using classical variables, were adjusted via quantization rules to make correct predictions. This approach worked up to a point but ran into major difficulties by the 1920s. By contrast, classical equations were used by Heisenberg as unaltered, which was unexpected at the time, but as explained below, was in effect a form of Bohr’s correspondence principle, a major tool of the old quantum theory.

Heisenberg’s new variables, however, were different and even more unexpected, as such variables had never been used in physics previously. They were (unbounded) infinite matrices, in effect operators in Hilbert spaces over ℂ. The Hilbert-space formalism of QM was introduced by von Neumann shortly thereafter, and was not known to Heisenberg, although he quickly adopted it (e.g., [10]). Heisenberg was famously unaware of the existence of matrix algebra. He reinvented it to find the variables he needed. Technically, while matrix algebra in finite and infinite dimensions was developed in mathematics by then, unbounded infinite matrices, used by Heisenberg and by Born and Jordan, had not been previously studied. As became apparent later, such matrices are necessary to derive the uncertainty relations for continuous variables. The multiplication of these matrices, which Heisenberg had to define as well to use his new variables in his old (formally classical) equations, is in general not commutative. This was another new feature of his theory, which was met with much suspicion, including, initially, by Heisenberg himself. As unbounded self-adjoint operators, these matrices do not form an algebra with respect to the composition as a noncommutative product, although some satisfy the canonical commutation relation. Most crucial, however, was that a purely abstract nature of this mathematics was only used to predict, with the help of additional rules (such as Born’s rule), the outcomes of experiments, rather than to represent physical objects and their behavior and make predictions based on such representations. The latter aim, which has defined all physics hitherto, was abandoned by Heisenberg, thus amounting to the introduction of Heisenberg discontinuity.

Defined over ℂ, these matrices as such could not represent anything observable, because observable physical quantities could only be represented by real numbers or functions of real numbers. (Technically, all measurable numbers are rational.) These matrices and then operators still came to be called “observables”, because they were linked, in terms of probabilistic predictions, to the corresponding physical quantities, such as position or momentum, observed in measuring instruments. To do so, one needed additional rules, such as Born’s rule, that allowed one to relate the complex quantities involved to positive real numbers that are probability densities associated with predictions concerning quantum events. (Heisenberg adopted this type of rule for a limited case of transitions between stationary states.) As a result, as emphasized in this article, the equations of QM were no longer equations of motions either, although they formally look like and have sometimes been (and still are) considered as such. They represented nothing physical in space and time. They were equations for probabilistic information transfer between measuring devices, which, along with this information itself, are described by classical physics. QM was conceived by Heisenberg as a new form of physics, insofar as these probabilistic connections of information found in measuring devices were not assumed to be underlain by the spacetime description of motion of atomic particles. In fact, that there was such a motion, however conceived, was not assumed either.

The correspondence principle, which, as initially understood by Bohr, required that the predictions of quantum theory must coincide with those of classical mechanics in the classical limit, was given a mathematical form by Heisenberg, making it the mathematical correspondence postulate. It stated that the equations and variables of QM convert into those of classical mechanics in the classical limit. Because Heisenberg formally retained the equations of classical physics, the first conversion was automatic. The second was not. The correspondence with classical theory could, however, still be maintained because Heisenberg’s new variables could be replaced by conventional classical variables in the classical limit. One could do so, for example, in the case of large quantum numbers, when the electrons were far away from the nuclei and when classical concepts, including orbits, could apply, although the electrons’ behavior was still quantum there and could have quantum effects. The interference of measuring instruments could be neglected as well.

Heisenberg added another twist: “What I really like in this scheme is that one can really reduce all interactions between atoms and the external world… to transition probabilities” (Heisenberg, Letter to Kronig, 5 June 1925; cited in [14] (v. 2, p. 242)). By speaking of the “interactions between atoms and the external world”, this statement suggests that QM was only predicting the effects of these interactions observed in measuring instruments. This view, not found in Bohr’s 1913 theory, was adopted by Bohr, to the point of becoming the defining feature of his interpretation in all of its versions, eventually compelling him to add Bohr discontinuity to Heisenberg discontinuity. It also led Bohr to replace the concept of measurement in the classical sense (that of measuring some pre-existing properties of quantum objects) with a new concept. A quantum measurement was now understood as establishing, by using measuring instruments, a quantum phenomenon, observed classically, without measuring the properties of quantum objects. In Bohr’s ultimate view, these properties were no longer assigned to them even at the time of measurement, rather than only apart from measurements. The classical treatment of the observable parts of measuring instruments meant that the data registered there, as part of quantum phenomena, could be measured as classical properties just as one measures such properties in classical physics. Measuring instruments were, however, also assumed to contain a quantum stratum through which they interacted, quantumly, with quantum objects. As quantum, this stratum and this interaction were equally placed beyond representation and, in Bohr’s ultimate interpretation, conception.

Heisenberg spoke of his new variables and the algebraic relationships between them as the “new kinematics”. This was not the best choice of term because, in contrast to Einstein’s kinematics of special relativity, Heisenberg’s new variables were not related to motion, as the term kinematic would suggest. As Einstein was to lament later, the theory was not even a mechanics, because it did not offer a representation of individual quantum processes. It only predicted, probabilistically or statistically, what was observed in measuring instruments. As such it was, for Einstein, akin to statistical physics. This assessment may, however, depend on how one understands mechanics as a mathematical-experimental theory. Bohr spoke of Heisenberg’s discovery as inaugurating “a new epoch of mutual stimulation of mathematics and mechanics” [9] (v. 1, p. 51). It was, however, no longer a mechanics of motion, which would represent a classically causal and ideally deterministic behavior of the ultimate individual constituents of matter. The quantum-mechanical predictions are probabilistic, regardless of how elemental the system considered is in any interpretation.

I have discussed how this new situation in fundamental physics was established in Heisenberg’s 1925 paper introducing QM on several previous occasions, most recently close to the present argument in [3] (pp. 112–122). I shall only state here the reasons, which must be apparent from the preceding discussion, for seeing—as I do—Heisenberg’s approach as quantum-informational in spirit, and conversely, quantum-information theory (in RWR-type interpretations) as Heisenbergian in spirit. The quantum-mechanical situation, as Heisenberg conceived of it, was defined by:

(A) certain already-obtained information, derived from spectral lines (due to the emission of radiation by the electron), observed in measuring instruments; and
(B) certain possible future information, to be obtainable from spectral lines to be observed in measuring instruments and, hopefully, predictable in probabilistic or statistical terms by the mathematical formalism of a quantum theory.

The term “information” is used here in the mathematical sense of information theory as digital information, a collection of classical bits. This is a specific type (devoid of semantic content) of information in more general sense of organized (in-*form*-ation) knowledge concerning something, although it may be given this more general meaning too. Heisenberg aimed at developing such a formalism, without assuming that it needed to represent a spatiotemporal process connecting these two sets of information or how each comes about, thus defining Heisenberg discontinuity [12] (p. 265). This information is, in each case, determined by what type of experiment one decides to perform, rather than by measuring pre-existing properties of quantum objects. Heisenberg’s theory was, thus, dealing with information obtainable in measuring instruments, physically described by classical physics, which at the same time could not predict this information.

The theory made both classical and quantum physics necessary in dealing with quantum phenomena, defined by classical information contained in them and quantum discontinuity. This discontinuity was manifested mathematically in the formalism of QM, as no longer representing how this information comes about. The mathematical formalism of quantum theory, QM or QFT, is abstract or, as Bohr would have it, symbolic. It is abstract in its literal sense (defining, as discussed above, modern mathematics) of divorcing mathematics from representing both material objects and objects represented by our general phenomenal intuition. It is symbolic because it uses formalism, such as the Hamiltonian one, that is formally analogous to that of classical mechanics, without, again, providing a physical representation of the reality considered, as classical mechanics does. It has no physical connection, beyond making probabilistic predictions of the outcomes of quantum experiments, to the ultimate reality responsible for quantum phenomena (Heisenberg discontinuity) or to anything observed as part of such phenomena (Bohr discontinuity). These phenomena are effects of the interactions between this reality and measuring instruments.

At one level, this character of QM and then QFT is not surprising. The formalism of QM or QFT, in all its standard versions so far, is defined over ℂ. By contrast, all data observed in physical phenomena and, presumably, whatever happens in space and time between them, would have to be represented by real quantities, as they are in classical physics or relativity. Both theories may use complex mathematical entities, but such entities are not essential in the way they are in quantum theory, because they do not define essential variables and are no longer contained in the solution of their equations, solutions representing the physical processes considered. The role of complex quantities in QM made Schrödinger lament the use of the complex wave function, his great invention. In introducing it, he characterized it as “extraordinarily convenient for the calculations”, while arguing that “the very much more congenial interpretation that the state of the [quantum] system [would be] given by a real function and its time derivative” [15] (p. 123). He hoped at the time that such a mathematical scheme over ℝ would eventually be possible; that was not to be, however. Instead, the formalism was soon supplemented by Born’s rule, which connected it to probabilities of quantum events, further supporting the essentially probabilistic nature of QM. This was not something that Schrödinger was happy about either. What was and remains surprising and enigmatic—*un*-reasonable—is that this scheme—combining or at least allowing some interpretations, those of the RWR type, to combine the Heisenberg and Bohr discontinuities—works. Bohr’s interpretation, especially in its ultimate version, gave a strong RWR-type form to Heisenberg discontinuity, and added Bohr discontinuity to it.

## 3. Bohr Discontinuity: From Heisenberg to Bohr, with Quantum Measurement

Bohr introduced his interpretation, along with his most famous concept, complementarity, in 1927 in the Como lecture [9] (v. 1, pp. 52–91). Intriguingly, the Como lecture retreated from a weak RWR-type view (adopted by Bohr in his initial response to Heisenberg’s discovery of QM) to a more realist view. Bohr, however, quickly abandoned his Como argument in favor of the RWR view, following his first exchange on the nature of QM with Einstein at the Solvay conference in Brussels only a month later. He developed his interpretation, via several revisions, culminating in his ultimate, strong RWR-type version during the next decade, in part under the impact of his continuing debate with Einstein. Bohr’s interpretation, in any of its versions, will be distinguished here from “the Copenhagen interpretation”, because there is no single such interpretation, as even Bohr has changed his a few times. I have considered different versions of Bohr’s interpretation previously [16] and shall only comment on them here as necessary for the present argument. While the differences—discontinuities—between them are important, there are also important continuities, in particular the irreducible role of measuring instruments in the constitution of quantum phenomena, a feature that was found in all of them and that eventually led Bohr to the Bohr discontinuity. In the final exposition of his interpretation in 1958, Bohr adopted the language of information in speaking of this role: “The quantal features of the phenomenon are revealed in the information about the atomic objects derived from the observations” [9] (v. 3, p. 4). It is conceivable that Bohr had in mind Shannon’s information theory, developed by then, although it is more likely that he used “information” (as he did previously) as referring to knowledge in general. Regardless of Bohr’s intended use, however, the statement allows one to see this information in information-theoretical terms as composed of classical units (bits). This information is obtained from classically defined measurements, but is predictable only by QM, or possibly another quantum theory, and not by classical physics.

The essential role of measuring instruments in the constitution of quantum phenomena was, however, brought into the foreground, beginning with the Como lecture, by way of a contrast with classical physics or relativity. In these theories, Bohr noted, “our … description of physical phenomena [is] based on the idea that the phenomena concerned may be observed without disturbing them appreciably” [9] (v. 1, pp. 53; emphasis added). By contrast, “any observation of atomic [quantum] phenomena will involve an interaction [of the object under investigation] with the agency of observation not to be neglected” [9] (v. 1, p. 54; emphasis added). My emphasis indicates a subtle nature of this contrast: the interaction between the object under investigation and the agency of observation gives rise to a quantum phenomenon rather than disturbs it. Bohr became weary of using the language of “disturbing of [quantum] phenomena by observation” [9] (v. 2, p. 64). At stake is instead the irreducible role of experimental technology in the constitution of quantum phenomena. Bohr’s statement does not imply—and Bohr never claimed so—that these phenomena are ever independent of our means of observing them, but only that they may be treated as such for all practical purposes within the proper scope of classical physics or relativity. Hence, Bohr speaks of “the idea” that these phenomena may be so observed, rather than stating that such is actually the case. Ultimately, the observation of any physical phenomenon involves an interaction between the world and our agencies of observation, beginning with our bodies. This interaction defines all physical phenomena, without allowing us to be certain that these phenomena or theories accounting for them represent, however ideally or approximately, nature as it exists independently. In classical physics or relativity, however, assuming that a theory does so is a practically justified and workable idealization. QM or QFT precluded this type of idealization in RWR-type interpretations, as based in the irreducible role of experimental technology in the constitution of quantum phenomena. Quantum phenomena were no longer assumed to be defined apart from human interaction with nature or by allowing one to neglect this interaction, as in classical physics or relativity—or so it appeared. Quantum physics helped us to realize that experimental technology, beginning, again, with that of our bodies, irreducibly shapes all physical phenomena, as discussed in detail in [3].

Of course, one can assume that phenomenal entities considered in physics could ground representations, ideal or approximate, of nature by means of physical theories, and thus allow one to form viable concepts of nature, or some parts of it, as an independent physical reality. However, as Kant had already realized, it is difficult to guarantee that such phenomenal entities—even the most basic ones, such as space, time, or motion, or our theories based on them—correspond to how nature ultimately is independently of us. A phenomenon may be assumed to represent or reflect a thing-in-itself, but any such assumption is always a construction that may not correspond to this thing-in-itself. This constructive character of our thought, even in the case of our immediate perceptions or what so appears (because it is now assumed to be mediated by conscious or unconscious thought) distinguished Kant’s philosophy from that of his predecessors. The RWR view is, however, more radical than that of Kant. While Kant defined things-in-themselves or noumena as not knowable, he allowed that they may be conceivable, even though there is no guarantee that such a conception is ultimately correct, even if it is workable in practice [2] (p. 115). What Kant called “reason” may be seen as allowing a greater—and in some interpretations of Kant, a full—guarantee of the truth of such a conception. By contrast, the strong RWR view places the ultimate character of the reality considered beyond conception. Kant’s view is closer to the weak RWR view, and it may appear to be the same. It may, however, be shown to be short of the weak RWR view and to remain a form of realism, moreover classically causal in character, which is expressly precluded by the RWR view [3] (pp. 57–58). In any event, at stake in the present argument is the strong RWR view.

The RWR view is still assumed here to be an idealization, and as such only as practically justified, given quantum phenomena and theories predicting them, such as QM or QFT. The RWR view is inferred from the character of quantum phenomena, as grounding their interpretation and quantum theory, rather than merely assumed on external philosophical grounds. (By idealization I refer to a workable conception of something or, as this is an idealization as well, the lack or impossibility of such a conception; in other words, something that may be different from what it idealizes rather than, as in some uses of the term, any form of approximation, defined by a proximity to what is idealized). Assuming that the RWR view is an idealization, ultimately only practically justified, also precludes one from definitively claiming that the ultimate constitution of the reality responsible for quantum phenomena is of the RWR type. This reality may be assumed, eventually even by those who had previously adopted the RWR view, to be ultimately conceivable or representable, in accord with one or another realist interpretation of quantum phenomena and QM or QFT, or whatever theory may replace it. There are realist views that exhibit affinities with Bohr’s argumentation, even if not his interpretation (e.g., [17,18] and further references there to related works by these authors).

Bohr’s ultimate interpretation was originally proposed by Bohr in his article “Complementarity and Causality” [19] and then discussed (without essential changes) in several subsequent communications, beginning with the Warsaw lecture [20]. Bohr does not use the language of reality without realism, but his understanding of the irreducible role of the interactions between quantum objects and measuring instruments clearly amounts to the strong RWR view. According to Bohr:

The renunciation of the ideal of causality in atomic physics which has been forced on us is founded logically only on our not being any longer in a position to speak of the autonomous behavior of a physical object, due to the unavoidable interaction between the object and the measuring instruments which in principle cannot be taken into account, if these instruments according to their purpose shall allow the unambiguous use of the concepts necessary for the description of experience.

[19] (p. 87)

The concept of causality that grounds this ideal of causality will be designated in this article as “classical causality”. I have several reasons for adopting this designation instead of just “causality”, as is more common in designating this concept, including by Bohr in most of his writings. (There is an important exception in one of his later works, when Bohr adopts a different view of causality [9] (v. 3, p. 4–5), which I shall discuss later). First, this concept has defined classical physics from its birth and was then extended to relativity, which, however, introduced certain restrictions on it. It has a much longer history in philosophy, going all the way to Plato or even the pre-Socratics. Secondly, there are alternative definitions of causality, including those probabilistic in nature, which classical causality is not, although it may be part of probabilistic theories, such as classical statistical physics or chaos theory. I shall discuss such definitions and propose one, “quantum causality”, later.

The concept of classical causality is defined by the assumption that the state (state X) of a physical system is determined, in accordance with a law, at all future moments of time once it is determined at a given moment of time (state A), and state A is determined in accordance with the same law by any of the system’s previous states. This assumption thus implies a concept of reality, which defines this law and makes this concept of causality ontological. By contrast, RWR-type interpretations are not classically causal because of the absence of realism, necessary for such a law, in considering the behavior of quantum objects. It is a more complex issue, whether A could be seen as the cause of X. The fact that it is not necessarily the case and related considerations has compelled some, beginning with P. S. Laplace, to speak of determinism in referring to this concept. I shall use the term determinism differently, as an epistemological category referring to the possibility of predicting the outcomes of classically causal processes ideally exactly. In classical mechanics, when dealing with individual objects or small systems, both concepts coincide or rather are correlative. On the other hand, classical statistical mechanics or chaos theory are not deterministic in this definition in view of the complexity of the systems considered, which limit us to probabilistic or statistical predictions concerning their behavior.

Bohr’s position, stated above, represents the strong RWR view, placing the ultimate nature of reality responsible for quantum phenomena beyond conception, a radical form of Heisenberg discontinuity; for if “due to the unavoidable interaction between the object and the measuring instrument”, we are no “longer in a position to speak of the autonomous behavior of a physical object”, this behavior must also be beyond conception. If we had such a conception, we would be able to say something about this behavior. It is true that there is a difference between forming some conception of this reality and forming a rigorous conception that would enable us to provide a proper representation of it. Bohr, however, clearly makes a stronger claim: we are no longer in a position to speak of the autonomous behavior of quantum objects at all. This view is confirmed by other key statements representing his ultimate view, such as “in quantum mechanics, we are not dealing with an arbitrary renunciation of a more detailed analysis of atomic phenomena but with a recognition that such an analysis is *in principle* excluded” [9] (v. 2, p. 62).

Given that there is no definitive statement to that effect on Bohr’s part, it is a matter of interpretation whether—in accord with two possibilities stated from the outset of this article—for Bohr, our inability to do so:

(A) characterizes the quantum-mechanical situation only as things stand now, while allowing that the quantum phenomena or whatever may replace them will no longer require this assumption and thus will make RWR-type interpretations no longer viable; or
(B) reflects the possibility that this reality will never become available to thought.

(B) might be suggested by some of his stronger statements, including in the 1937 article under discussion. In any event, in his ultimate interpretation, Bohr at least adopts (A), although my argument here would apply if he adopted (B). As explained earlier, the qualification “as things stand now” still applies to (B). It applies not because the unthinkable nature of the ultimate constitution of the reality responsible for quantum phenomena becomes available to thought, which it cannot under (B). It applies because a return to realism in quantum theory or what might replace it is possible. This would make (B), or (A), obsolete even for those who held it previously.

Bohr’s ultimate interpretation was grounded, along with complementarity (adjusted to this interpretation), in a new concept: that of phenomena, defined strictly in terms of effects, observed in measuring instruments as a result of their interaction with quantum objects. As explained earlier, in Bohr’s ultimate interpretation, a quantum measurement is not a measurement of some preexisting property of a quantum object. Instead, it establishes, creates, a quantum phenomenon, the observed physical properties of which can then be measured classically. According to Bohr:

I advocated the application of the word phenomenon exclusively to refer to the observations obtained under specified circumstances, including an account of the whole experimental arrangement. In such terminology, the observational problem is free of any special intricacy since, in actual experiments, all observations are expressed by unambiguous statements referring, for instance, to the registration of the point at which an electron arrives at a photographic plate. Moreover, speaking in such a way is just suited to emphasize that the appropriate physical interpretation of the symbolic quantum-mechanical formalism amounts only to predictions, of determinate or statistical character, pertaining to individual phenomena appearing under conditions defined by classical physical concepts [describing the observable parts of measuring instruments].

[9] (v. 2, p. 64)

Referring to “observations” is precise, because only the classically observed properties of measuring instruments could be measured. (In this article, by “quantum measurement” I refer to this whole process.) As defined by “the observations [already] obtained under specified circumstances”, phenomena are associated only with events that have already occurred and not with possible future events. Referring, phenomenologically, to observations also explains Bohr’s choice of the term “phenomenon”. This “idealization of observation” is the same as that of classical physics [9] (v. 1, p. 55). It allows one to identify phenomena, described by classical physics, with the physical objects, which objects are now measuring instruments themselves, because our observation does not interfere with their behavior. On the other hand, measuring instruments do interfere with quantum objects or the ultimate constitution of the reality responsible for phenomena.

Quantum discreteness also becomes that of phenomena, rather than the Democritean atomic discreteness of the ultimate constitution of the RWR-type reality responsible for quantum phenomena or of quantum objects [9] (v. 2, pp. 32–34). Quantum objects are now placed beyond conception and hence cannot be seen either as continuous or discrete. While central at the earlier stages of the debate concerning quantum theory, the role of discreteness in quantum theory in general has been a somewhat neglected subject in recent foundational discussions, with only a few exceptions (e.g., [17] and further references there). This role was, of course, unavoidably prominent in commentaries on Bohr. Around the time when he introduced his concept of phenomena, Bohr also introduced a concept of “atomicity”, pertaining to quantum phenomena rather than quantum objects [9,20] (v. 2, p. 34; p. 94). This concept is essentially equivalent to that of phenomena but highlights some key aspects of the latter [16] (pp. 138–150). It transfers to the level of observable phenomena, manifested in measuring instruments, the key “atomic” features of quantum physics—discreteness, discontinuity, individuality, and atomicity (as indivisibility)—previously associated with quantum objects. “Atomicity” in Bohr’s sense is thus a feature of physically complex and divisible entities, and not of physically indivisible entities, such as elementary particles. Bohr discontinuity is related to Bohr’s concept of atomicity because it dissociates the formalism of QM, which predicts the information obtained in phenomena, from the classical physical description of these phenomena and this information.

In Bohr’s ultimate interpretation, giving Heisenberg discontinuity a radical form, no physical quantities are assumed to correspond to properties of quantum objects even at the time of measurement. This is the case even in considering single properties, rather than only certain joint properties, not attributable simultaneously by virtue of the uncertainty relations. Bohr’s earlier views allowed for this type of attribution at the time of measurement, under the constraints of the uncertainty relations, thus still in accord with the assumption that such properties cannot be considered independently of measurement. In Bohr’s ultimate interpretation, however, an attribution even of a single property to any quantum object is never possible—before, during, or after measurement. Even when we do not want to know, say, the momentum of a quantum object, and thus need not worry about the uncertainty relations, the position of this object itself is never determinable, and only the position of the corresponding effect, such as a spot on a photographic plate, is. The uncertainty relations remain valid, of course, as do other standard physical laws such as conservation laws, but they now apply to the corresponding (classical) variables of the observed parts of measuring instruments impacted by quantum objects. One can either prepare our instruments to be able to measure a change in the momentum of certain parts of them or to locate the spot that registers an impact of a quantum object, but never both. The uncertainty relations are correlative to the mutually exclusive nature of these arrangements, in accord with complementarity, as explained in detail below. In the interpretation adopted in this article, even a quantum object, such as an electron, is, again, an idealization applicable only at the time of measurement, rather than independently, as in Bohr’s ultimate interpretation, although a quantum object is assumed to be beyond conception in both interpretations.

Bohr’s insistence on the indispensability of classical physical concepts in considering measuring instruments and defining their observable part has often been misunderstood, beginning with disregarding that measuring instruments contain both classical and quantum strata. Classical concepts only represent the observable parts of measuring instruments. The instruments must, however, be able to interact with quantum objects—or, in the present view, the ultimate constitution of the reality responsible for quantum phenomena—for these phenomena to be possible. Any such interaction requires a quantum stratum in the constitution of measuring instruments. This interaction is quantum and cannot be observed, or in RWR-type interpretations, represented. It is “irreversibly amplified” to the classical level of observable effects, say, a spot left on a silver screen (e.g., [9] (v. 2, p. 73)). According to Bohr:

It would be a misconception to believe that the difficulties of atomic theory may be evaded by eventually replacing the concept of classical physics by new conceptual forms. Indeed, … the recognition of the limitation of our forms of perception by no means implies that we can dispense with our customary ideas and their direct verbal expression when reducing our sense of impressions to order. No more is it likely that the fundamental concepts of the classical theories will ever become superfluous for the description of physical experience. The recognition of the indivisibility of the quantum of action, and the determination of its magnitude, not only depend on an analysis of measurements based on classical concepts, but it continues to be the application of these concepts alone that makes it possible to relate the symbolism of the quantum theory to the data of experience. At the same time, however, we must bear in mind that the possibility of an *unambiguous* use of these fundamental concepts solely depends upon the self-consistency of the classical theories from which they are derived and therefore, the limits imposed upon the application of these concepts are naturally determined by the extent to which we may, in our account of the phenomena, disregard the element which is foreign to classical theories and symbolized by the quantum of action.

[9] (v. 1, p. 16)

Bohr’s argument is, thus, not that old concepts such as those of classical physics are sufficient to offer a representation of the behavior of quantum objects. His argument is that no concepts—old or new—could do so, while old concepts—specifically those of classical physics—are essential for handling quantum phenomena observed in measuring instruments. At the same time, these old concepts are not sufficient for doing so, for example, because of complementarity, or again, for allowing one to predict what is so observed. New physical concepts are still possible in quantum theory, and Bohr’s concepts, such as complementarity and phenomenon, are among them. Their structure, as defined by the RWR view, is different, however: it combines “old” classical concepts and that which is beyond concepts. Classical physical concepts are only part of the overall conceptual structure of Bohr’s concepts of phenomenon and complementarity. They are used to describe the physics of the observable parts of measuring instruments. In this respect, classical concepts are, again, indispensable. Because, however, no concept of any kind could apply to the ultimate constitution of the reality responsible for quantum phenomena, the need for new concepts to represent this reality would be meaningless in Bohr’s interpretation.

In his later book *Physics and Philosophy*, Heisenberg addressed the key paradox (or what so appears) at the heart of “the Copenhagen interpretation”: “[The Copenhagen interpretation] starts from the fact that we describe our experiments in the terms of classical physics and at the same time from the knowledge that these concepts do not fit nature accurately. The tension between these two starting points is the root of the statistical character of quantum theory” [11] (p. 56). “The Copenhagen interpretation” presented in the book is a mixture of Bohr’s and Heisenberg’s own, in some respects, different views. (Heisenberg’s views there are helpfully discussed in Ref. [21].) On the point in question at the moment, however, Heisenberg’s position is fully in accord with, and in fact follows, that of Bohr:

It has sometimes been suggested that one should depart from the classical concepts altogether and that a radical change in the concepts used for describing the experiments might possibly lead back to a nonstat[ist]ical [sic!], completely objective description of nature. … This suggestion, however, rests upon a misunderstanding. The concepts of classical physics are just a refinement of the concepts of daily life and are an essential part of the language which forms the basis of all natural science. Our actual situation in science is such that we do use the classical concepts for the description of the experiments, and it was the problem of quantum theory to find theoretical interpretations of the experiments on this basis. There is no use in discussing what could be done if we were other beings than we are. At this point we have to realize, as von Weizsäcker has put it, that “Nature is earlier than man, but man is earlier than natural science”. The first part of the sentence justifies classical physics, with its ideal of complete objectivity. The second part tells us why we cannot escape the paradox of quantum theory, namely, the necessity of using classical concepts.

[11] (p. 56)

Classical physical concepts reflect those of human thinking as defined by our biological and specifically neurological machinery, born with our evolutionary emergence as human animals; the point, again, often made by Bohr, as considered by this author [3] (pp. 64–65) and other commentators (e.g., [22]). Any concept we form derives from and applies only to observed phenomena, and quantum phenomena are physically classical as observed phenomena. They are different from classical phenomena because the data observed in them prevents us, at least in RWR-type interpretations, from describing how they come about by classical physics or, in these interpretations, by quantum theory. Quantum theory, however, can probabilistically predict these data without describing or representing how they come about, which defines Heisenberg discontinuity. It follows that the formalism of QM has no connection to the classical description of the observed parts of measuring instruments and phenomena in Bohr’s sense. This disconnection adds Bohr discontinuity to Heisenberg discontinuity, further grounding the latter, by splitting quantum and classical theory, while making both necessary, including in quantum theory itself.

The role of Bohr discontinuity is easy to underappreciate or miss, which can lead to misunderstanding of Bohr’s concept of phenomena and his interpretation. It is, accordingly, worth considering it further. Bohr discontinuity implies that the mathematical symbols comprising the formalism of QM have no connections to the physics of quantum phenomena observed in measuring instruments and described by classical physics and its concepts or symbols. Classical concepts and symbols of classical theories do describe—or more accurately, ideally represent—the behavior of the observable parts of measuring instruments. By contrast, by Bohr discontinuity, the symbols of the formalism of QM, as a purely symbolic theory, only relate to the probabilities of the outcomes of quantum experiments, with the help of Born’s rule, added as a separate postulate. Thus, contrary to some contentions (e.g., [23]), the symbols of QM do not in any way represent our physical encounters with measuring instruments. It is mistaken to assume, as in [23] (p. 30), that these symbols have anything to do with, in Bohr’s words, “what we have done and what we have learned” in quantum experiments in considering which “the account of the experimental arrangement and the results of the observation must be expressed in unambiguous language with suitable application of the terminology of classical physics” [9] (v. 2, p. 34).

The symbols of the formalism of QM can be unambiguously communicated as well, as can be any mathematical symbols. However, they have no connection with the results of the observation, as communicated between human agents “in unambiguous language with suitable application of the terminology of classical physics”. While enabling probabilistic predictions concerning the information contained there, these symbols, by Bohr discontinuity, have no connection with the (classical) physical nature of these observations. It is essential for Bohr’s interpretation that QM only predicts, by using its symbols, the probabilities or statistics of the outcomes of possible future experiments and does not relate to what has already happened. What has been observed or measured is independent of QM and is represented by classical physics. As Bohr said: “There can be no question of any unambiguous interpretation of the symbols of quantum mechanics other than that embodied in the well-known rules which allow us to predict the results to be obtained by a given experimental arrangement described in a totally classical way” [24] (p. 701). Obviously, contrary to [23], as such, these symbols in no way represent the classical and in part daily-language or narrative accounts of “what we have done and what we have learned” [9] (v. 2, p. 39). These accounts only pertain to the outcomes of quantum experiments as phenomena and the information contained there, now specifically in mathematical sense of information theory. These data can be communicated unambiguously as, in this sense, objective facts [9] (v. 2, pp. 68–69, v. 3, p. 7). So can be, as a mathematical fact, the formalism of QM, giving this formalism an objective content as well, while still allowing it to be physically dissociated from these classical data. This dissociation defines Bohr discontinuity, coupled to Heisenberg discontinuity in Bohr’s interpretation.

As noted, Bohr discontinuity may apply in the absence of Heisenberg discontinuity. For example, it would apply if one assumed, as Heisenberg eventually did, that the formalism of QM (or QFT) represents the ultimate constitution of physical reality in the absence of physical concepts as ordinarily understood (as in classical physics and relativity). Bohr discontinuity would still apply, because the phenomena observed and measurements performed—and hence any physical support of information thus obtained—would still be handled classically, just as it would be if one assumes Heisenberg discontinuity.

There may of course be human ingredients, based on or defining information in its broader sense of human knowledge, that each of us brings to observing quantum phenomena and dealing with the information (in its information-theoretical sense) registered in measuring instruments. Our interpretations of quantum phenomena or our theories, such as QM, and our decisions to accept or reject such interpretations depend on such ingredients. Bohr recognizes this. He does say that “the appropriate widening of our conceptual framework [in either relativity or QM] [does not] imply any appeal to the observing subject, which would hinder unambiguous communication of experience” (Bohr 1987, v. 3, p. 7). To say, however, that a conceptual framework of a theory does not imply any appeal to the observing subject does not mean that there are no observing subjects or that these subjects do not, individually or collectively, play roles in using this framework or in experiences associated with it. Their presence is clearly implied by the reference to “communication of experience”. Whose experience, or a communication between whom, would it be otherwise? It is an experience of a human subject communicated, by means of language (involving but not limited to technical terminology) to other human subjects. Bohr often refers to “experience” rather than only to an experiment, including in several passages cited by this article (e.g., Bohr 1937, p. 87, Bohr 1987, v.1, pp. 12–18, v. 2, p. 57, v. 3, p. 7). These are not casual uses of the word. Bohr’s uses of any words are rarely casual.

This communication must, however, be unambiguous for the conceptual framework used to conform to the requirement of modern physics as a mathematical-experimental science of natural phenomena, which are experienced by human agents. While we, as humans, use physical theories and assign probabilities (possibly being affected by more personal aspects of our thinking), we must share their verification for a physical theory, such as QM, to be a mathematical-experimental science. In this regard, the mathematical conceptualization of information, a form of mathematical reduction of information, by information theory, from Shannon on, eventually leading to quantum information theory, was decisive. Making Shannon the Galileo of information theory, his mathematization of information may be compared to the Galilean reduction in physics (from Aristotelian physics) that made it mathematical-experimental science. In retrospect, this was also a reduction of human information (as knowledge in general) to mathematical information, or mathematized physical information. Modern physics was an information theory all along.

Science is a human enterprise, and as such inevitably involves extrascientific elements of human experience, individual and collective. However, sharing and communicating our estimates of possible events and experiences is also human; and doing so is not only helpful but is also unavoidable in human life. Science capitalizes on this fact and on the possibility that the communication involved may be made unambiguous, helped by using mathematical symbols, central to modern physics from Galileo on. As Bohr says: “Just by avoiding the reference to the conscious subject which infiltrate daily language, the use of mathematical symbols secures the unambiguity of definition required for objective [unambiguously communicable] description” (Bohr 1987, v. 2, p. 68). This statement confirms that for Bohr, mathematical symbols do not represent “classical physical explanation… of what we have done and what we have learned”, which would entail a reference to the conscious subject and additional explanations by using daily language, even in the mathematical aspects of physics. This is the case even in classical physics and relativity, but in quantum physics, in Bohr’s view, combining Heisenberg and Bohr discontinuity, the use of mathematical symbols has no representational connections to observations possible in classical physics and relativity.

I consider next Bohr’s concept of complementarity from this perspective, which defines this concept in Bohr’s ultimate interpretation. As defined most generally, complementarity is characterized by:

(A) a mutual exclusivity of certain phenomena, entities, or conceptions; and yet
(B) the possibility of considering each one of them separately at any given point; and
(C) the necessity of considering all of them at different moments of time for a comprehensive account of the totality of phenomena that one must consider in quantum physics.

The concept was never given by Bohr a single definition of this type. However, this definition may be surmised from several of Bohr’s statements, such as: “Evidence obtained under different experimental conditions cannot be comprehended within a single picture, but must be regarded as complementary in the sense that only the totality of the phenomena [some of which are mutually exclusive] exhaust the possible information about the objects” [9] (v. 2, p. 40; emphasis added). In classical mechanics, we can comprehend all the information (either in the sense of information theory or more generally) about each object within a single picture because the role of measurement in the constitution of physical phenomena can be disregarded. This allows one to identify the phenomenon considered with the object under investigation and to establish the quantities defining this information, such as the position and the momentum of each object, in the same experiment. In quantum physics, this interference cannot be disregarded and defines any quantum phenomenon. This leads to different experimental conditions for each measurement on a quantum object (assumed to be prepared in some way, by a previous event) and their complementarity, in correspondence with the uncertainty relations. The situation implies two incompatible pictures of what is observed, as phenomena, in measuring instruments. Hence, the possible information about a quantum object—the information to be found in measuring instruments—could only be exhausted by the mutually incompatible evidence obtainable under different experimental conditions. On the other hand, once made, either measurement—say, that of the position—will provide the complete actual information about the system’s state, as complete as possible, at this moment in time. One could never obtain the complementary information, provided by the momentum measurement, at this moment in time. To do so, one would need simultaneously to perform a complementarity experiment on it, which is never possible. Both types of information can *never* be obtained in a single-measurement situation.

It follows that one cannot assume that two complementary measurements represent parts of the same whole, of the same single reality. Each measurement establishes the only reality there is, and the alternative decision would establish a different reality, at all three levels of idealization, assumed in this article: the ultimate nature of the reality responsible for quantum phenomena, quantum objects, and quantum phenomena, even though in the first two cases this reality is each time unknowable and even unthinkable. It may still be assumed to be each time different because each of its effects, observed as a phenomenon, is different. Rather than arbitrarily selecting one or other part of a pre-existing physical reality, as is ideally possible in classical physics, our decisions concerning which experiment to perform establish the single reality which defines what type of quantity can be observed or predicted and precludes the complementary alternative. Accordingly, parts (B) and (C) of the above definition of complementarity are as important as part (A) and disregarding them can lead to a misunderstanding of Bohr’s concept. That we have a free (or at least sufficiently free) choice of what kind of experiment we want to perform is in accordance with the very idea of experimentation in all science, including in classical physics [24] (p. 699). However, in quantum physics, implementing our decision will allow us to make only certain types of predictions and will irrevocably exclude certain other, complementary, types of predictions.

It may be noted that wave–particle complementarity, with which the concept of complementarity is often associated, had not played a significant, if any, role in Bohr’s thinking, especially after the Como lecture. Bohr thought deeply, even before QM, about the problem of wave–particle duality, as it was known then. Bohr was, however, always aware of the difficulties of applying the concept of physical waves to quantum objects or of thinking in terms of the wave–particle duality as the assumption that both types of nature and behavior pertain to the same individual entities considered independently. The wave–particle duality was thought of as representing the same thing in two different ways. By contrast, complementarity refers to two different, indeed incompatible, things. The “both” (both types of properties) of the wave–particle duality is the opposite of complementarity, based on “either or”, which is difficult, if possible at all, to apply to quantum objects. Bohr’s ultimate solution to the dilemma of whether quantum objects are particles or waves was that they were neither. Either “picture” may refer to one of the two mutually exclusive sets of discrete individual effects of the interactions between quantum objects and measuring instruments. These effects may be particle-like, which may be individual or collective, or wave-like, which are always collective, but are still composed of discrete individual effects. An example the latter is provide by “interference” effects, composed of a large number of discrete traces of the collisions between the quantum objects and the screen in the double-slit experiment in the corresponding setup, when both slits are open and there are no means to know through which slit each object has passed. Such effects are no longer observed in any setup when such a knowledge is at least possible. These two sets of effects, both properly predicted by QM, are complementary: they are mutually exclusive and require mutually exclusive experimental setups to be observed, while we can always decide on either setup. They are, however, not an instance of wave–particle complementarity, except in a metaphorical sense.

In Bohr’s post-Como argumentation, complementarity becomes primarily exemplified by complementarities of spacetime coordination and the application of momentum or energy-conservation laws, correlative to the uncertainty relations. They are complementarities of phenomena observed in measuring instruments, and thus in effect in accord with Bohr’s concept of phenomena, introduced a decade later. Technically, the uncertainty relations, Δ*q*Δ*p* ≅ *h* (where *q* is the coordinate, *p* is the momentum in the corresponding direction), only prohibit the simultaneous exact measurement of both variables. This is always possible, at least ideally and in principle, in classical physics, and allows one to maintain classical causality there. The physical meaning of the uncertainty relations is, however, much deeper in Bohr’s interpretation and a complex subject in its own right with a long history (e.g., [25,26,27], and for a view close to that of Bohr [28,29] (p. 93; p. 133)). A few essential points could be noted here, however.

First, the uncertainty relations are not a manifestation of the limited accuracy of measuring instruments, because they would be valid even if we had perfect instruments. In classical and quantum physics alike, one can only measure each variable within the capacity of our measuring instruments. In classical physics, however, one can in principle measure both variables simultaneously within the same experimental arrangement and improve the accuracy of this measurement by improving capacity of our measuring instruments, in principle indefinitely. The uncertainty relations preclude us from doing so for both variables in quantum physics regardless of this capacity. Each type of measurement is complementary to the other, which by the definition of complementarity given above, also means that each by itself could be measured ideally exactly. According to Bohr: “we are of course not concerned with a restriction as to the accuracy of measurement, but with a limitation [by complementarity] of the well-defined application of space-time concepts and dynamical conservation laws, entailed by the necessary distinction between measuring instruments and atomic objects” [9] (v. 3, p. 5). In Bohr’s view, moreover, one not only cannot measure both variables simultaneously but also cannot define them simultaneously. It follows that one cannot define such elements alternatively for the same quantum object. One always needs two objects to define both variables. Thus, if, after determining, the position of the electron, emitted from a source, at time *t_m_* after the emission, we want to determine the momentum, we need to repeat the same identically prepared emission of another electron and then measure its momentum after the same time interval *t_m_* after the emission.

Probabilistic or statistical considerations are unavoidable in considering both the uncertainty relations, which is self-evident, and complementarity, which is not and is often overlooked. The essentially probabilistic nature of quantum predictions is, however, equally reflected in complementarity, which Bohr saw as a “generalization of the very ideal of causality” [9] (v. 2, p. 41). Bohr never explained what he had in mind by this generalization. It can, however, be understood in terms the concept of quantum causality, probabilistic in nature, which is anticipated in Bohr’s arguably final (and the shortest) exposition of his interpretation, given in *Causality and Complementarity*, published in 1958 [9] (v. 3, pp. 1–7). The article contains, it appears for the first and only time in Bohr’s writing, a view of causality applicable in quantum physics, as opposed to classical causality to which Bohr had previously referred by the term causality. Bohr says:

Although, of course, the classical description of the experimental arrangement and the irreversibility of the recordings concerning the atomic objects ensure a sequence of cause and effect conforming with elementary demands of causality, the irrevocable abandonment of the ideal of determinism finds striking expression in the complementary relationships governing the unambiguous use of the fundamental concepts on whose unrestricted combination the classical physical description rests. Indeed, the ascertaining of the presence of an atomic particle in a limited space-time domain demands an experimental arrangement involving a transfer of momentum and energy to bodies such as fixed scales and synchronized clocks, which cannot be included in the description of their functioning, if these bodies are to fulfil the role defining the reference frame. Conversely, any strict application of the law of conservation of momentum and energy to atomic processes implies, in principle, a renunciation of detailed space-time coordination of the particles.

[9] (v. 3, pp. 4–5)

There is no conflict here with Bohr’s previous appeal to the renunciation of “the classical ideal of causality”, as classical causality, because the view of causality suggested here is not classical. This renunciation is now replaced with “the irrevocable abandonment of the ideal of determinism”, which is in effect the same ideal, because in considering individual or simple classical systems both ideals coincide. It remains crucial that this epistemological scheme is possible by virtue of “the classical description of the experimental arrangement and the irreversibility of the recordings”, with the mathematical formalism of QM, obeying Bohr discontinuity. The irreversibility of the recordings is found in classical physics and relativity as well, where, however, it obeys classical causality. On the other hand, while—given an earlier comment in the article [9] (v. 3, p. 4)—Bohr must have had in mind the connections between causality, thus understood, and probability, he only speaks of “ensur[ing] a sequence of cause and effects conforming with the elementary demands of causality”, rather than defining a concept of causality that conforms to these demands. Quantum causality, as a form of probabilistic causality, is such a concept. It was introduced by this author previously [3,29,30], which, especially [3] (pp. 207–211), my discussion here follows.

Quantum causality is defined as follows. An actual event that has happened determines which events may or (in view of complementarity) may not happen and be predicted with one probability or another, which is not the same as saying that any of them will happen. This event, A, at time t_0_ defines certain possible, but only possible future events, say, X, at time t_1_. In the case of individual or simple (nonchaotic) classical or relativistic systems, probabilistic causality reduces to classical causality, and correlatively, determinism. The temporal precedence of A and the corresponding (local) arrow of time is crucial, and I shall further discuss it below. It follows, however, that in contrast to classical causality, quantum causality defines such future events without definitively establishing—or rather (because it would be established in advance), being definitively related to—any future state of the system considered. This is because one can, at t_1_, perform an alternative, complementary measurement and thus, by an alternative decision, establish an event (Y) and a reality different from the one predicted (X) even if this prediction was made with a probability of one, as in the case of the EPR-type experiments. It follows that such predictions are only meaningful if X is still possible, a point to which I shall return presently. Only the temporal precedence of A vis-à-vis either X or Y is definitive. Quantum causality allows one to relate actual events in terms of statistical correlations, such as, again, those of the EPR-type between them, the events of which are, however, specifically prepared by repeated initial measurements. No definitive relationships between any two actual events—events that have already occurred—could be established, even ideally, as they can always be in classical mechanics. There these events are defined by quantities that pre-exist measurement rather than being established by measurement, under the conditions of complementarity and the uncertainty relations, as in quantum physics in RWR-type interpretations. Hence, there is no probability (except equal to one or zero) in classical mechanics of individual or simple systems, while there is always probability, in general not equal to one, in quantum physics, no matter how simple the system considered.

The concept of quantum causality gives a meaning (one possible meaning) to Bohr’s view of complementarity as a generalization of causality—of “the very ideal of causality” [9] (v. 2, p. 41). On the one hand, “our freedom of handling the measuring instruments, characteristic of the very idea of experiment” in all physics—our “free choice” concerning what kind of experiment we want to perform—is essential [24] (p. 699). On the other hand, as against classical physics or relativity, implementing our decision concerning what we want to do will allow us to make only certain types of predictions and will exclude the possibility of complementary types of predictions. Complementarity thus generalizes causality, because it defines which events can or cannot be probabilistically predicted by our decision concerning which experiment to perform, without establishing future events as bound to happen, which excludes classical causality.

In addition to Bohr’s 1958 anticipation cited above, several concepts of quantum, or more generally, probabilistic causality, were proposed in quantum information theory (e.g., [31,32,33]). M. D’Ariano defines causality in general (at least in physics) by means of this type of concept [33]. D’Ariano’s argument places a strong emphasis on the arrow of time, within a framework somewhat different from the one proposed below. Classical causality or, for individual or small classical systems, correlatively, determinism (to which D’Ariano primarily refers) would then be just a special case of causality in this probabilistic sense. The concept of quantum causality proposed here brings new dimensions to the question of causality in quantum theory and quantum information theory. It does so because the (strong RWR-type) interpretation that is adopted here and that underlies this concept redefines the relationships between quantum information and quantum discontinuity, by combining the Heisenberg and Bohr discontinuities. This combination also has important implications for the question of temporality, which I shall now consider.

I note first that, while one can speak of quantum causality or the related concepts just mentioned, it is difficult to speak of “causes”. Consider two quantum events: A, as the initial measurement (preparation); and X, as the second measurement, predicted with a given probability by QM on the basis of A. First of all, one cannot, even ideally or in principle (as in classical physics or relativity), be certain that X will happen in quantum physics. More importantly, as indicated earlier, even when our prediction concerning X can be made, at time t_0_, on the basis of the measurement A, with (ideally) probability one, as in EPR-type experiments, this probability assignment is conditional: it is only meaningful if the measurement defining X is performed or at least is possible to perform. One can, however, always make an alternative, complementary, measurement at time t_1_ which will disable the original prediction and define a different event or reality Y at t_1_ [3] (pp. 210–212). One cannot circumvent this difficulty by assuming that the system considered can simultaneously possess both quantities in the way it would in classical physics, or Bohmian mechanics, thus ensuring classical causality there. One cannot see A as the cause of Y either because Y is not an observation with the preparation A, as X would be. However, once Y is defined, X is no longer possible. All these events or the corresponding phenomena, A, X, and Y, are effects of the interactions, at the time of measurement, between measuring instruments and quantum objects. One cannot, however, say that our preparation of measuring instruments is a cause of an “effect” observed either. This is not the case because any measurement outcome is defined by the interaction between the object and the instruments rather than by this preparation, and as such is never guaranteed either. Neither event, A, X, or Y, may happen in any given case.

What always remains in place is the temporal precedence of A relative to either X or Y, or whatever else happens at time of the second measurement. Either measurement or event, X or Y, would respect this precedence, as it cannot precede A, and thus would respect the arrow of time. For the reasons just explained, however, the arrow of time, along with quantum causality, is, in RWR-type interpretations, only manifested classically in observable phenomena. What can, in these interpretations, be objectively ascertained is that the ultimate nature of reality responsible for quantum phenomena is such that all our interactions with it, on all scales, by means of experimental technology entail the arrow of time. Quantum causality only manifests the arrow of time in the case of quantum phenomena. This need not mean that at the ultimate level of reality responsible for quantum phenomena, there is no change or multiplicity but only permanence and oneness, as is sometimes suggested (e.g., [34], although J. Barbour adjusted his view in [35]). In (strong) RWR-type interpretations, this concept would not apply to the ultimate constitution of reality any more than those of change or motion, or any other concepts, such as space or time, and thus, the arrow of time. While each time unknowable or even unthinkable, the ultimate nature of the reality responsible for quantum phenomena manifests itself differently in its effects observed in quantum phenomena.

By the same token, the equations of QM or QFT, such as Schrödinger’s or Dirac’s equation, are not equations of the motion of quantum objects, which, moreover, are only defined at the time of measurement in the interpretation adopted here. These equations only provide (with the help of Born’s rule), in Schrödinger’s apt language, “expectation-catalogs” for the outcomes of future experiments, which implies the arrow of time [36] (p. 154). Accordingly, their formal time reversibility, sometimes used to argue against the arrow of time at the ultimate level of reality, has no physical significance. In fact, what is reversible is not *time* but the value of the parameter t, associated with possible future measurements, which respect the arrow of time as, to return to Bohr’s phrasing, “the irreversibility of the recordings” [9] (v. 3, p. 4). This irreversibility is, again, found in all physics, in RWR-type interpretations predicted, under the condition of quantum discontinuity, combining Heisenberg and Bohr discontinuity. Physical equations, whether classical, relativistic, or quantum, only make predictions concerning events in time, the sequence of which as actual events is not reversible or have never been shown to be thus far. This complicates the assumption of time-reversibility even in classical physics and relativity, in which cases, too, what is reversible is the parameter *t*, associated with possible measurements, which would, as far as we know, always respect the arrow of time (e.g., [3] (pp. 215–217)). This view also precludes the concept of the so-called Block Universe (at least in its conventional form, roughly, as an unchanging four-dimensional block of spacetime, vs. a three-dimensional space governed by the passage of time) and, against it, reestablished both causality and the arrow of time (e.g., [33]). The Block Universe is, by definition, a realist conception, and is automatically excluded by RWR-type interpretations of quantum theory, or by extending RWR view to all physics, including relativity, with which the concept of the Block universe originates. This extension is discussed by this author in [3]. One does not need the RWR view to maintain the arrow of time in quantum theory, one just needs it in classical physics and relativity, commonly interpreted in realist terms. The differential equations of classical physics or relativity can, however, be assumed, for all practical purposes, to represent the continuity of events in time, even when the arrow of time is assumed, which is not so for the differential equations of QM or QFT in RWR-type interpretations. Such interpretations only assume the existence of discrete events and make the mathematics of these equations relate such events, without, by Heisenberg discontinuity, physically connecting them, and, by Bohr discontinuity, physically representing them. This representation is left to classical physics, thus making both classical and quantum theory necessary in dealing with quantum phenomena.

## 4. Conclusions: How Many Theories?

The concept of Bohr discontinuity amplified a radical understanding of the nature of fundamental physics brought about by the concept of Heisenberg discontinuity in quantum physics. It also revealed the unavoidable role of both classical and quantum physics in quantum theory and arguably in all fundamental physics, with relativity added. Relativity is, however, analogous to classical physics, insofar as both are essentially “based on the idea that the phenomena concerned may be observed without disturbing them appreciably”, an idea no longer applicable in quantum physics [9] (v. 1, p. 53). Relativity is also a classically causal and indeed deterministic theory. By contrast, QM and QFT are not, although they allow for probabilistic forms of causality, as considered above.

The heterogeneity of fundamental physics, brought about by Bohr difference, did not help, but, if anything, exacerbated the common epistemological discontent with QM or QFT. This discontent was created by the difficulty, or even impossibility, of a realist—and preferably classically causal—theory of the ultimate constitution of the physical reality responsible for quantum phenomena. To have two types of fundamental theories—or with relativity, three—physically, mathematically, and philosophically different from each other was not welcome news either, especially in the context of the unification programs, on which I shall comment presently. I hasten to add, however, that this situation also does not help the view that all physical phenomena could be ultimately handled by one form or another of quantum theory. This view is not uncommon, for example, among the proponents of the many-worlds interpretation of QM. On the other hand, the situation is in harmony with an informational approach to fundamental physics because it can ground different types of theories, such as classical and quantum theory (e.g., [37]).

J. S. Bell’s view is a paradigmatic example of this discontent with a heterogeneity of fundamental physics, conjoined with the epistemological discontent with the nonrealism of QM. Bell expressly refers to Bohr in this connection. While giving Bohr credit for clarifying this situation, Bell criticizes Bohr, quite severely, for going beyond a merely pragmatic use of both classical and quantum theory and making this split to define fundamental physics [38] (pp. 188–190). Like Einstein, Bell would have preferred all fundamental physics to be of the same type, ideally classical-like, on the model of classical mechanics or relativity. Indeed, the (1986) article in question ultimately champions “the pilot wave picture” (even while recognizing the lack of a proper theory corresponding to it), as “professional”, vs. the “romantic” alternative of the Copenhagen and expressly Bohr’s view. Bell says: “We could also consider how our possible worlds [worldviews] of physics measure up to professional standards. In my opinion the pilot wave picture undoubtedly shows the best craftsmanship among the pictures we have considered. But is that a virtue in our time?” [38] (p. 195). Putting aside a somewhat sanctimonious moral sentiment, this is a rather idiosyncratic view. “Undoubtedly”? In 1986? Would extraordinary developments of QFT, leading to the discoveries of quarks, electroweak bosons, and for forth (including at CERN, were Bell was during those decades) not be any less provisional? None of them were based on the pilot wave theory. Furthermore, why were Heisenberg’s and Bohr’s “pictures” any less professional? For the moment, the division of “the world” into two equally bothers Bell, as does the shiftiness of “the cut”, fully consistent with the present view [3] (pp. 65–76). Bell says:

Thus, in contemporary quantum theory that the world must be divided into a wavy ‘quantum system’, and a remainder which is in some sense ‘classical’. The division is made one way or another, in a particular application, according to the degree of accuracy and completeness aimed at. For me it is the indispensability, and above the shiftiness, of such a division that is the big surprise of quantum mechanics. It introduces an essential ambiguity into fundamental physical theory, if only at the level of accuracy and completeness beyond any required in practice. It is the tolerance of such an ambiguity, not merely provisionally but permanently, and at the most fundamental level, that is the real break with the classical ideas. It is this rather than the failure of any particular concept such as ‘particle’ or ‘determinism’.

[38] (p. 188)

The phrase “an essential ambiguity” is borrowed from Bohr (e.g., [38] (p. 155)). Bohr invokes it on several occasions, especially in his reply to EPR [24]. In part in referring to his reply to EPR, he speaks of “the essential ambiguity involved in a reference to physical attributes of objects when dealing with phenomena where no sharp distinction can be made between the behavior of the objects themselves and their interaction with the measuring instruments” [9] (v. 2, p. 61). The absence of this distinction is equivalent to Heisenberg discontinuity. Bell bypasses the fact that this shiftiness has a limit, a fact not missed by Bohr. While, as Bohr observes, “it is true that the place within each measuring procedure where this discrimination [between the object and the instrument] is made is… largely a matter of convenience”, it is true only largely, but not completely. This is because “in each experimental arrangement and measuring procedure we have only a free choice of this place within a region where the quantum-mechanical description of the process concerned is effectively equivalent with the classical description” [24] (p. 701).

Bell follows by assessing (again, critically) Bohr’s “complementarity” as “one of the romantic worldviews inspired by quantum theory. It emphasized the bizarre nature of the quantum world, the inadequacy of everyday notions and classical concepts. It lays stress on how far we have left behind naïve 19th century materialism” [38] (p. 190). Bell wants none of this. He offers a possible—and to him, preferable—alternative:

Suppose that we accept Bohr’s insistence that the very small and the very big be described in very different ways, in quantum and classical terms. But suppose we are skeptical about the possibility of such a division being sharp and above all about such a possibility being shifty. Surely the big and the small should merge smoothly with one another? And surely in fundamental physical theory this merging should be described not just in vague words but precise mathematics? This mathematics would allow electrons to enjoy the cloudiness of waves, while allowing tables and chairs, and ourselves, and black marks on photographs, to be rather definitively in one place rather than another and to be described ‘classical terms’. The necessary technical theoretical development involves introducing what is called ‘nonlinearity’ and perhaps what is called ‘stochasticity’ into ‘Schrödinger’s equation’. There have been interesting pioneering effort in this direction, but not yet a breakthrough. The possible way ahead is unromantic in that it requires mathematical work by theoretical physicists, rather than an interpretation by philosophers, and not the promise lesson in philosophy for philosophers.

[38] (p. 190)

Technically, in Bohr’s view, QM does not, by Heisenberg discontinuity, describe the word in the very small, but only predicts its effects on the (macroworld) world we observe. There is a question of the very big, too, which or the *very* very big, may require a theory different from any available theory we have, classical, quantum, or relativistic. One can assume, however, that Bell refers to the daily-life scale of classical physics. Again, however, was Heisenberg’s creation of QM not a professional work, even one of the best ever by a theoretical physicist, grounded as it was in Heisenberg discontinuity? On the other hand, is not a picture of “electrons … enjoy[ing] the cloudiness of waves” romantic, even naively, anthropomorphically, romantic? Would Heisenberg’s realization that “fortunately, mathematics is not subject to this limitation” of daily concepts and pictures, such “the cloudiness of waves”, a freedom that allowed him to create QM, not be more professional [10] (p. 11)?

The alternative proposed by Bell could not of course be excluded. How likely is it, however? “Surely the big and the small should merge smoothly with one another?” Bell says. The question mark may well be more fitting than “surely”. One might prefer this alternative, but one cannot be sure that it will emerge. It remains possible that two—or with relativity, three, or more—different types (physically, mathematically, and philosophically) of fundamental theories will be necessary for moving fundamental physics forward.

This situation also makes one—at least this author—question the programs or problems of unification in fundamental physics, such as, prominently, the following three:

(1) The problem of the quantum-to-classical transition in quantum theory. This problem is not commonly considered under the heading of unification (often by assuming that the ultimate constitution of reality is either quantum or classical-like), but is in effect a problem of unification, and programs aiming to resolve it, such as decoherence, are unification programs.
(2) The problems of the “grand unified theory” (GUT) of the standard model of elementary particle physics. The standard model is comprised of three forms of QFT: that of electromagnetic interactions, quantum electrodynamics (QED), with the local gauge symmetry group U(1); that of weak interaction, with the local gauge symmetry group SU(2); and the strong nuclear interaction, quantum chromodynamics (QCD), with the local gauge symmetry group SU (3). The first two are unified in the electroweak theory, with GUT as a possible—but not yet available—unification of all three into a single QFT, with one large gauge symmetry and several force carriers, with one unified coupling constant. Most proposals predict the existence of new particles with extremely high masses, beyond the reach of currently envisioned collider experiments, at the GUT scale, 10^16^ GeV, below the Planck scale, 10^19^ GeV, and thus, if they exist, only inferable indirectly.
(3) The third is the problem of a theory, sometimes referred to as a theory of everything (TOE). Such a theory would unify electronuclear interactions, currently presumed quantum, and gravity, which currently has no quantum form, but is governed by Einstein’s general relativity (GR). As things stand now, GR and QFT are inconsistent with each other, while the experimental data at stake in both theories are consistent. TOE need not be quantum, although many assume that is likely to be, with some form of GUT being a prerequisite for TOE.

In the present author’s (probabilistic) estimate, (2) is the only one that is likely to succeed, as a quantum theory. This is still not certain, first because we might still have several different quantum theories in place (that is, the electroweak theory and QCD may not be unified); and secondly, because it may still happen that on some scale such a theory will no longer be quantum or will only be partly quantum in the present sense. Neither (1), in view of Bohr discontinuity, nor (3) are likely in my estimate. Besides, if Bohr’s discontinuity still holds, “TOE” will not be a theory of everything. I am not saying that they are impossible, let alone not desirable, which would be plainly incorrect because each, or (2), is desired by many. Nor can one deny the history of successful unifications in physics, beginning with that of electromagnetism, via the concept of electromagnetic field. It served as the main inspirations for Einstein’s and related, such as Weyl’s, relativity-based unification projects. I am only saying that these unifications may not be possible.

I would also argue that neither of these unifications may be necessary for the advancement of fundamental physics. This advancement is of course possible and necessary, for example, in developing gravity theory on the microscale of the current (Fermi) scale of QFT or beyond, say, on the Planck scale. Such a theory may, however, not be quantum in the current sense. It may also not emerge as a unification of all fundamental forces and be a separate theory, thus proving that the advancement of fundamental physics need not depend on unification projects.

We may not exist in many worlds (such as those of the many-worlds interpretation of QM), and I do not think we do; however, we may well live in a world of many physical theories, including fundamental ones, existing in multiple continuities, discontinuities, and interactions with each other. It is difficult to predict where fundamental physics will find itself in the future. It is also possible that we will discover theories very different from any we have now, or even unimaginable, for example, theories that are neither realist nor of the RWR type. Such a theory is difficult to imagine, given that the strong RWR view assumes that the ultimate constitution of the reality responsible for quantum phenomena or the ultimate constitution of nature in general is beyond thought. What, then, could an alternative be apart from one or another form of realism, if defined, as here, by assuming that the reality considered is at least conceivable? One might, however, still be reluctant to exclude this possibility. Nobody had expected or imagined anything like the physical reality that relativity or quantum physics made it possible to think, and yet we—at least, some of us—came to think of either form of reality not only as possible but also as likely.
